# Antibody-mediated delivery of a viral MHC-I epitope into the cytosol of target tumor cells repurposes virus-specific CD8^+^ T cells for cancer immunotherapy

**DOI:** 10.1186/s12943-022-01574-0

**Published:** 2022-04-22

**Authors:** Keunok Jung, Min-Jeong Son, Se-Young Lee, Jeong-Ah Kim, Deok-Han Ko, Sojung Yoo, Chul-Ho Kim, Yong-Sung Kim

**Affiliations:** 1 Department of Allergy and Clinical Immunology, Ajou University School of Medicine, Suwon, 16499 Republic of Korea; 2 Department of Molecular Science and Technology, Ajou University, 206 Worldcup-ro, Yeongtong-gu, Suwon, 16499 Republic of Korea; 3 Department of Otolaryngology, Ajou University School of Medicine, Suwon, 16499 Republic of Korea

**Keywords:** MHC-I epitope cytosolic delivery, Cytosol-penetrating antibody, Peptide–MHC-I complex, Anti-viral cytotoxic T lymphocytes, Cytomegalovirus therapeutic cancer vaccine

## Abstract

**Background:**

Redirecting pre-existing virus-specific cytotoxic CD8^+^ T lymphocytes (CTLs) to tumors by simulating a viral infection of the tumor cells has great potential for cancer immunotherapy. However, this strategy is limited by lack of amenable method for viral antigen delivery into the cytosol of target tumors. Here, we addressed the limit by developing a CD8^+^ T cell epitope-delivering antibody, termed a TEDbody, which was engineered to deliver a viral MHC-I epitope peptide into the cytosol of target tumor cells by fusion with a tumor-specific cytosol-penetrating antibody.

**Methods:**

To direct human cytomegalovirus (CMV)-specific CTLs against tumors, we designed a series of TEDbodies carrying various CMV pp65 antigen-derived peptides. CMV-specific CTLs from blood of CMV-seropositive healthy donors were expanded for use in in vitro and in vivo experiments. Comprehensive cellular assays were performed to determine the presentation mechanism of TEDbody-mediated CMV peptide-MHC-I complex (CMV-pMHCI) on the surface of target tumor cells and the recognition and lysis by CMV-specific CTLs. In vivo CMV-pMHCI presentation and antitumor efficacy of TEDbody were evaluated in immunodeficient mice bearing human tumors.

**Results:**

TEDbody delivered the fused epitope peptides into target tumor cells to be intracellularly processed and surface displayed in the form of CMV-pMHCI, leading to disguise target tumor cells as virally infected cells for recognition and lysis by CMV-specific CTLs. When systemically injected into tumor-bearing immunodeficient mice, TEDbody efficiently marked tumor cells with CMV-pMHCI to augment the proliferation and cytotoxic property of tumor-infiltrated CMV-specific CTLs, resulting in significant inhibition of the in vivo tumor growth by redirecting adoptively transferred CMV-specific CTLs. Further, combination of TEDbody with anti-OX40 agonistic antibody substantially enhanced the in vivo antitumor activity.

**Conclusion:**

Our study offers an effective technology for MHC-I antigen cytosolic delivery. TEDbody may thus have utility as a therapeutic cancer vaccine to redirect pre-existing anti-viral CTLs arising from previously exposed viral infections to attack tumors.

**Supplementary Information:**

The online version contains supplementary material available at 10.1186/s12943-022-01574-0.

## Background

Cancer immunotherapy based on cytotoxic CD8^+^ T lymphocyte (CTL)-mediated tumor recognition and elimination has shown remarkable anticancer efficacy [1]. Nonetheless, the suppression or absence of antitumor CTLs in the tumor microenvironment (TME) and tumor immune escape (evasion of an antitumor CTL response) limit the clinical efficacy in many patients with solid tumors [1, 2]. CTLs recognize cancer cells through the T cell receptor (TCR) specific to tumor-derived antigenic peptides presented by major histocompatibility complex class I (MHC-I; HLA class I in humans) [2]. Personalized therapeutic cancer vaccines are designed to deliver tumor-specific T cell epitopes, so-called neoantigens, in various forms (synthetic long peptides, mRNA, and DNA) with the intent of inducing de novo tumor-specific CTL responses and/or amplifying the endogenous tumor-specific CTL responses [1–3]. This approach has shown promising antitumor activity in early clinical trials for melanoma and other cancers [2, 4]. However, some of the challenging aspects of neoantigen-based cancer vaccines include the difficulty in identifying and selecting immunogenic neoantigens, the downregulation or loss of the MHC-I alleles on tumor cells, the poor homing of neoantigen-specific CTLs into TME, and their personalized nature, which may not be suitable for all patients [1–3]. Remarkably, antiviral CTLs specific to human viruses (that have previously infected the host), such as human cytomegalovirus (CMV), Epstein–Barr virus, and influenza virus, have been found to infiltrate various solid tumors abundantly [5, 6]. The tumor-infiltrating antiviral CTLs cannot attack tumor cells because of the lack of specific recognition of the tumor through the TCR. Nevertheless, repurposing of some antiviral bystander CTLs arising from common human viral infections to attack tumors holds great potential for cancer immunotherapy due to their abundance, high potency, and specificity to common viruses [7].

To utilize pre-existing antiviral CTLs to attack tumors, tumor cells should first be marked with a viral peptide–MHC-I complex (pMHCI) on the cell surface, to be recognized by the antiviral CTLs. To this end, some approaches, such as extracellular surface loading [6, 8–10] and endosomal loading [11] of a viral CTL epitope peptide onto MHC-I, have been explored. All the existing strategies deviate from the conventional MHC-I antigen presentation pathway [12], wherein cytosolic localization of viral antigens is critical for intracellular processing intended to form mature MHC-I epitopes (8–11 amino acid residues in length) for efficient binding to MHC-I in the endoplasmic reticulum (ER), followed by surface presentation in the form of pMHCI. Few studies have addressed the delivery of MHC-I-restricted CTL epitopes into the cytosol of tumor cells, mainly due to the dearth of efficient cytosolic delivery tools. To overcome this limitation, we envisioned that a tumor-specific cytosol-penetrating antibody (Ab) that we recently documented, dubbed the inCT cytotransmab [13–15], can serve as a carrier for cytosolic delivery of MHC-I-specific antigenic peptides into target tumor cells. In human immunoglobulin G1 (IgG1/κ) format, inCT can access the cytosol of target tumor cells after endocytotic internalization via a tumor cell-associated receptor (integrin αvβ3 or αvβ5; mainly integrin αvβ5 on epithelial cancer cells), followed by endosomal escape into the cytosol [13–15].

In this study, we sought to direct CMV-specific CTLs against tumors because CMV infection is very common among healthy adults (60–90% of the population, with higher infection rates with increasing age) [16] and is characterized by accumulation and maintenance of CMV-specific CTLs with a majority of effector memory phenotypes: a phenomenon termed memory inflation [17, 18]. In CMV-seropositive hosts, the 65 kDa phospho-protein (pp65) antigen-derived 9-mer peptide ^495^NLVPMVATV^503^ (residues 495–503, hereafter referred to as the “CMVp_495–503_ peptide”) is the most immunogenic CTL epitope among CMV antigens [19] and is predominantly displayed on HLA-A*02:01 [20, 21], which is the most prevalent MHC-I variant in the human population [22]. CTLs specific to the CMVp_495–503_–HLA-A*02:01 complex (hereafter referred to as “CMV-pMHCI”) are present in the blood with high prevalence and functional competence among both HLA-A*02:01^+^ healthy donors and cancer patients [9, 23, 24]. CMV-pMHCI-specific CTLs (hereafter referred to as “CMVp-CTLs”) efficiently kill tumor cells displaying CMV-pMHCI on the surface [25], and therefore, are an attractive tool for cancer immunotherapy.

Here, to deliver a viral MHC-I epitope peptide into the cytosol of target tumor cells for converting them into virally infected cells, we engineered a CD8^+^ T cell epitope-delivering Ab, termed a TEDbody, by genetic fusion of the viral MHC-I antigen peptide to the inCT cytotransmab. The TEDbody efficiently delivered the payload of a CMVp_495–503_-encompassing peptide into the target tumor cells, such that it was intracellularly processed for surface presentation by the cognate MHC-I, thereby rendering the marked tumor cells recognizable and killable by the corresponding antiviral CMVp-CTLs. Furthermore, we showed that systemic injection of a TEDbody carrying the CMVp_495–503_-encompassing peptide into immunodeficient mice bearing preestablished human tumor xenografts recruits adoptively transferred CMVp-CTLs into the tumor, thus substantially inhibiting tumor growth. The potency of this approach was further augmented when combined with a CTL-stimulating anti-OX40 agonistic Ab.

## Methods

### Cell lines

The human cell lines [breast adenocarcinoma (MDA-MB-231 cells), colorectal carcinoma (HCT116 and LoVo cells), and small cell lung cancer (NCI-H889 cells)] were purchased from the Korean Cell Line Bank (Korea) and maintained in RPMI 1640 (HyClone, Korea). HEK293FT cells purchased from Invitrogen were maintained in Dulbecco’s modified Eagle’s medium (DMEM, Hyclone). All cells were cultured in a growth medium that was supplemented with 10% heat-inactivated fetal bovine serum (FBS; HyClone), penicillin (100 U/ml), streptomycin (100 μg/ml; Welgene), and amphotericin B (0.25 μg/ml; HyClone). All cell lines were authenticated by DNA short tandem repeat profiling (ABION CRO, Korea) and used within 10 passages. All the cell lines were maintained at 37 °C in a humidified 5% CO_2_ incubator and were routinely screened for *Mycoplasma* contamination (CellSafe, Korea).

### Reagents and abs

The peptides used in this study were synthesized (> 95% purity) by AnyGen (Gwangju, Korea) and are listed in Table S1 with the sequence information. Chemicals, protein reagents, and Abs used as reagents in this study are listed in Table S2.

### Expression and purification of TEDbodies and abs

An inCT light chain (LC) expression plasmid, pcDNA3.4–CT05-LC, encoding integrin αvβ5/αvβ3-targeting in4 cyclic peptide–CT05 light chain variable domain (VL)–Ck constant-domain sequence (residues 108–214 in EU numbering) has been described before [14]. An inCT heavy chain (HC) expression plasmid, pcDNA3.4-CT60 heavy chain variable domain (VH)–HC, carrying the human IgG1 constant domain sequence (CH1-hinge-CH2-CH3, residues 118–447 in EU numbering) with LALAPG mutations (L234A/L235A/P329G) in the CH3 domain has been described in previous studies [13, 14]. Regarding the endosomal escape motif-deficient inCT (AAA) Ab, inCT LC and HC expression plasmids carrying either CT05-AAA VL with a replacement of ^92^WYW^94^ by ^92^AAA^94^ or CT60-AAA VH with a replacement of ^96^WYW^98^ by ^96^AAA^98^ were used [13, 14]. For plasmids of peptide-fused Abs, including TEDbodies, DNA encoding the peptide and a G_4_S linker was subcloned in-frame without additional sequences at the C-terminus of the HC of each Ab. Expression plasmids for the anti-EGFR Ab necitumumab [26] and TCR-like Ab C1–17 [27] have been described previously. For the anti-programmed cell death protein 1 (PD1) Ab pembrolizumab (Keytruda®) and anti-OX40 (CD134) 1166/1167 Abs, DNA fragments of VH and VL of pembrolizumab (DrugBank Accession No. DB09037) and the 1166/1167 clone (patent WO 2018/202649) [28] were synthesized by Integrated DNA Technologies, Inc. (USA) and subcloned in-frame into a pcDNA3.4-based vector to be expressed in human IgG4 (with S228P)/κ and IgG1 (with LALAPG)/κ form, respectively. All the constructs were confirmed by sequencing (Macrogen, Korea).

For the expression of TEDbodies and Abs, the plasmids encoding HC and LC were transiently cotransfected in pairs at an equimolar ratio into cultured HEK293F cells in the FreeStyle 293F medium (Invitrogen) following the standard protocol [14]. Culture supernatants were collected after 6 to 7 d by centrifugation and filtration (0.22 μm, polyethersulfone; Corning, CL S43118). TEDbodies and Abs were purified from the culture supernatants on a protein A–agarose chromatographic column (GE Healthcare) and extensively dialyzed to switch the solution to histidine buffer (25 mM histidine, pH 6.5, 150 mM NaCl) [14, 29].

The plasmids for expression of interleukin-15 (IL-15)/IL-15 receptor α chain (IL-15Rα)-Fc, i.e., the human IgG1 Fc-fused Sushi domain of IL-15Rα complexed with IL-15 via a disulfide bond, were generated as previously described [30], where IL-15/IL-15Rα-Fc is reported as P22339. Briefly, DNAs encoding the IL-15 with the L52C mutation and the Sushi domain of IL-15Rα with the S40C mutation were synthesized (Bioneer, Korea) and then subcloned in-frame into the pcDNA3.4 vector and pcDNA3.4-human IgG1 HC Fc vector (hinge-CH2-CH3, residues 118–447 in EU numbering) with LALAPG mutations in the CH3 domain, respectively. IL-15/IL-15Rα-Fc was expressed by transient cotransfection of the two plasmids into cultured HEK293F cells; then, it was purified on a protein A–agarose chromatographic column and dialyzed against a final buffer [Dulbecco’s phosphate-buffered saline (PBS), pH 7.4].

Before cell treatment, all the purified TEDbodies, Abs, and proteins were sterilized using a cellulose acetate membrane filter (0.22 μm; Corning) and Mustang Q membrane filter (0.8 μm; Pall, MSTG25Q6). Protein concentration was determined with the Bicinchoninic Acid (BCA) Kit (Thermo Fisher Scientific) and by measuring the absorbance at 280 nm using the molar extinction coefficient calculated from the primary sequence.

### Cell line construction

To generate transporter associated with antigen processing 1 (TAP1) knockout MDA-MB-231 cells, a modified all-in-one lentiviral vector eSpCas9-LentiCRISPIR v2-TAP1 (Gene script)—containing a TAP1-targeting single guide RNA (sgRNA) sequence, enhanced green fluorescent protein (EGFP) (GenBank Accession No. AAB02572), and *Streptococcus pyogenes* Cas9 with mutations K848A, K1003A, and R1060A for enhanced target specificity (eSpCas9)—served as the selection marker. Three specific sgRNA target sequences (5′-CCCAGATGTCTTAGTGCTAC-3′, 5′-ACCTGTAGCACTAAGACATC-3′, and 5′-GGTGCGAGGCCTATGTCTCT-3′) for the TAP1 knockout were selected for this experiment. To create MDA-MB-231 cells stably expressing EGFP (MDA-MB-231-EGFP), the *EGFP* gene was inserted into lentiviral expression plasmid pLJM (Addgene), yielding pLJM-EGFP. For lentivirus production, 1 μg of the eSpCas9-LentiCRISPIR v2-TAP1 plasmid or pLJM-EGFP vector mixed with packaging plasmids (pMDLg/pRRE, pRSV/REV, and pMD2-G; Addgene) in an equimolar ratio was cotransfected into 10^6^ cells of HEK293FT producer cell line (Invitrogen) in 6-well plates (Corning) using Lipofectamine 3000 (Invitrogen), as previously described [29]. The viral supernatant was harvested at 48 h and passed through a 0.45-mm filter (Corning). MDA-MB-231 cells were transduced with each type of lentiviral particles in the presence of 6 μg/ml polybrene, and then, EGFP-expressing MDA-MB-231 cells were sorted on a FACS Aria III system (BD Biosciences) every 2 weeks for 2 months, to isolate TAP1 knockout MDA-MAB-231 cells or MDA-MB-231-EGFP cells. PD1- and OX40-expressing HEK293FT cells, named 293FT-PD1 and 293FT-OX40, respectively, were obtained by transient transfection of HEK293FT cells with either the pCMV3-PD1 plasmid (GenBank Accession No. NM_003327.2) or pCMV-OX40 plasmid (GenBank Accession No. NM_005018.2) from Sino Biological Inc. using Lipofectamine 3000 (Invitrogen) for 48 h.

### Peripheral blood mononuclear cell (PBMC) preparation and ex vivo expansion of CMVp-CTLs

PBMCs from healthy donors were acquired using protocols approved by the Institutional Review Board of Ajou University (approval ID: 201602-HM-001-01). All donors provided written informed consent before blood collection into a BD Vacutainer (BD Biosciences, 367,874). PBMCs were isolated using Ficoll-Paque Plus (GE Healthcare, 17–5442-03) density gradient centrifugation [29]. For long-term storage, PBMCs were resuspended with 10% FBS in DMSO and stored in liquid nitrogen at 1–5 × 10^6^ cells/ml [9]. For in vitro expansion of CMVp-CTLs, 4 × 10^6^ PBMCs (2 × 10^6^ cells/ml) were stimulated for 3 d at 37 °C with 5 μg/ml CMVp_495–503_ peptide in medium A [X-vivo medium supplemented with 2% of heat-inactivated human serum (Sigma)] in a 14-ml U-bottom tube (SPL). Then, the PBMCs were cultured for 3 d at 37 °C in medium B [medium A plus 200 IU/ml IL-2 (Peprotech)]. Next, the PBMCs were cultured every 2 to 3 d in medium C (medium B plus 0.5 nM IL-15/IL-15Rα-Fc protein) for up to 2 weeks. To evaluate the prevalence and phenotype of CMVp-CTLs, PBMCs before and after the ex vivo expansion were monitored by flow cytometric analysis involving double staining with a monoclonal Ab specific to CD8α (HIT8a) and a phycoerythrin (PE)-conjugated CMVp_495–503_-HLA-A*02:01 pentamer. At least two hundred thousand events were collected using a FACSCalibur flow cytometer (Becton Dickinson). Only PBMCs containing > 50% of CMVp-CTLs among all ex vivo-expanded PBMCs were used in in vitro cytotoxicity assays and in vivo adoptive transfers. The E:T ratio was calculated based on the prevalence of ex vivo-expanded CMVp-CTLs among the PBMCs.

### Flow cytometry

To detect CMV-pMHCI complex formed on the cell surface at 4 °C, the indicated cells (4 × 10^5^) were incubated at 4 °C for 3 h with a synthetic peptide, TEDbody, or control Ab at the indicated concentrations. For the detection of CMV-pMHCI on the cell surface at 37 °C, the indicated cells (1.5 × 10^5^) were seeded in a 12-well plate in the medium containing 10% FBS and cultured until they were fully attached to the bottom of the plates (~ 12 to 15 h). Next, the cells were treated for 18 h with a synthetic peptide, TEDbody, or control Ab at the indicated concentrations, washed with PBS, and stained with the CMV-pMHCI-specific C1–17 Ab (10 nM) [27] in a blocking solution [PBS (pH 7.4) and 2% FBS] for 1 h at 4 °C. After a wash with the ice-cold blocking solution, the cells were stained with an Alexa Fluor 647–labeled rabbit anti-mouse IgG Ab (Thermo Fisher Scientific) for 30 min at 4 °C. After a wash with 1 ml of ice-cold PBS, twenty thousand events were collected using the FACSCalibur flow cytometer. Flow cytometric data were analyzed using the FlowJo v10 software (Tree Star) to calculate mean fluorescence intensity (MFI) and geometric MFI (gMFI). Fold changes in the gMFI of CMV-pMHCI presentation on the cell surface were calculated by normalization to that of the cells stained solely with the secondary Ab.

To identify the memory phenotype of CMVp-CTLs and determine the cell surface expression of PD1, OX40, LAG-3, TIGIT, and CXCR3 on CMVp-CTLs, the cells were stained with fluorescently labeled primary Abs specific to CD45RA, CCR7, PD1, OX40, LAG-3, TIGIT, CXCR3, or the respective isotype control Abs [31]. At least two hundred thousand events were collected using the FACSCalibur flow cytometer. The cell surface expression of HLA-A*02, integrin αvβ3, integrin αvβ5, and PD-L1 on human cancer cells, as well as the binding ability of pembrolizumab (Keytruda®) and anti-OX40 1166/1167 Abs toward HEK293FT-PD1 and HEK293FT-OX40, were determined using the respective primary Ab or isotype control Abs and then an appropriate secondary Ab, according to the standard protocol [29, 31, 32]. For intracellular staining of IFN-γ, cells were activated with phorbol 12-myristate 13-acetate (100 ng/mL) plus ionomycin (500 ng/mL) in a humidified incubator with 5% CO_2_ at 37 °C for 10 h and further incubated for 6 h with brefeldin A (BD Biosciences, 1:1000) to prevent protein transport from the ER to the Golgi apparatus. All intracellular staining was performed using the BD Cytofix/Cytoperm Kit (BD Biosciences, cat. # 554714). Twenty thousand events were collected using the FACSCalibur flow cytometer. All flow cytometry experiments were performed at least three times independently, the data were analyzed using the FlowJo v10 software, and representative data are shown, unless otherwise stated.

### In vitro tumor cell lysis by ex vivo-expanded CMVp-CTLs

To evaluate the lysis of TEDbody-treated target cells by ex vivo-expanded CMVp-CTLs, cancer cells (5 × 10^3^) were seeded in a 96-well plate in the medium containing 10% of FBS and cultivated at 37 °C and 5% CO_2_ until they were fully attached to the bottom of the plates. After 12 to 15 h, the cells were treated with a synthetic peptide, TEDbody, or control Ab at the indicated concentrations for 12 h, washed with the medium, and cocultured for 18 h with ex vivo-expanded CMVp-CTLs at an E:T ratio of 5:1 (unless specified otherwise). In experiments with inhibitors, the cells were pretreated for 1 h with MG132 (Thermo Fisher Scientific) or cotreated for 8 h with either ERAP1-IN-1 (Chem Scene) or brefeldin A and with a synthetic peptide, TEDbody, or control Ab. Supernatants were employed to assess target cell lysis by lactate dehydrogenase (LDH) measurement and to evaluate T cell activation by an IFN-γ secretion assay. The LDH release was measured using the Cyto96 Non-Radio Cytotoxicity Assay (Promega) [29]. Absorbance was read at 492 nm using a Cytation 3 imaging multimode reader (Biotek). The maximum LDH release was determined by lysing the target cells with 1% Triton X-100 (Promega). The percentage of tumor cell lysis was calculated according to the following formula [29, 32]: tumor cell lysis (%) = 100 × [(LDH release with peptide or TEDbody or control Ab treatment minus spontaneous LDH release of target cells)/(maximum LDH release minus spontaneous LDH release of target cells)]. IFN-γ secretion into the supernatant was determined using the ELISA Ready-SET-GO Kit (Thermo Fisher Scientific). Absorbance was read at 450 nm on the Cytation 3 imaging multimode reader.

### Real-time cell lysis assays

Real-time kinetics of cell lysis were examined under a Lionheart FX automated microscope (BioTek Instruments) equipped with full temperature and CO_2_ control to maintain 37 °C and 5% CO_2_. Ex vivo-expanded CMVp-CTLs were stained with red fluorescent dye PKH26 (Sigma-Aldrich). MDA-MB-231-EGFP cells (5 × 10^3^) were seeded in a 96-well black clear-bottom plate (Greiner) in the medium containing 10% of FBS and cultured at 37 °C and 5% CO_2_ until they were fully attached to the bottom of the plates. After 15 h, the cells were treated for 12 h with 1 μM synthetic peptide, TEDbody, or control Ab, washed with the medium, and cocultured with PKH26-labeled CMVp-CTLs at an E:T ratio of 3:1 inside the Lionheart FX automated microscopy system. Images were captured every 1 h for up to 14 h in triplicate via a 10× objective. All cells were photographed in the bright-field channel, MDA-MB-231-EGFP cells in the GFP channel, and PKH26-labeled CMVp-CTLs were photographed in the TRITC channel. Nine photos per well were taken and stitched to cover the center of the well [23]. Quantification of fluorescence intensity from the total area of MDA-MB-231-EGFP cells was performed in the Gen5 software (BioTek). To adjust the data for differences in the initial cell number across the wells, fluorescence intensity from the total cancer cell area at each time point was normalized to that at the initial time point (0 h). This normalized fluorescence intensity from the total cancer cell area is referred to as the cell index in this article.

### Confocal immunofluorescence microscopy

Intracellular CMV-pMHCI induced by a TEDbody was visualized by confocal microscopy with a CMV-pMHCI-specific C1–17 Ab that was conjugated with DyLight 550 using the DyLight 550 Ab Labeling Kit (Thermo Fisher Scientific) [14]. Briefly, MDA-MB-231 cells (5 × 10^4^) grown on cell culture slides (SPL) were treated with a TEDbody or a control Ab for 18 h. After two washes with PBS, the cells were fixed with 2% paraformaldehyde in PBS for 10 min at 25 °C, permeabilized with 0.1% Triton X-100 in PBS for 10 min at 25 °C, blocked with 2% bovine serum albumin in PBS for 1 h at 25 °C, and then incubated with the DyLight 550-labeled C1–17 Ab (20 nM) for 1.5 h at 25 °C to stain intracellular CMV-pMHCI. Early endosomes and the Golgi apparatus were also stained with a mouse anti-early endosome antigen 1 (EEA1) Ab and a mouse anti-FTCD (58 K-9) Golgi protein Ab, respectively, for 1.5 h at 25 °C [33]. Next, the cells were washed and further incubated with a goat anti-mouse Ab conjugated with Alexa Flour 488 (secondary Ab; Thermo Fisher Scientific) for 1.5 h at 25 °C. Nuclei were stained with Hoechst 33342 in PBS for 5 min at 25 °C. After mounting of the coverslips onto glass slides with the Fluorescence Mounting Medium (Dako), center-focused single z-section images were captured using a Zeiss LSM 710 system with the ZEN software (Carl Zeiss).

### Mice

All animal experiments were approved by the Animal and Ethics Review Committee of Woojung Bio Inc. (Suwon, Korea) and performed in accordance with the guidelines established by the Institutional Animal Care and Use Committee. The approval ID for using the animals was IACUC2003–004 at the Animal Facility of Woojung Bio. Immunodeficient NSG mice (NOD.Cg-*Prkdc*^*scid*^*IL2rg*^*tm1Wjl/*^SzJ) were originally obtained from the Jackson Laboratory and bred and maintained at the Animal Facility of Woojung Bio. Female C57BL/6 mice were purchased from Orient Bio (Korea) and allowed to reach 5–6 weeks of age before tumor inoculation.

### In vivo CMV-pMHCI presentation and activation of tumor-infiltrating CMVp-CTLs

Female NSG mice (4–6 weeks old) received an orthotopic injection of MDA-MB-231 cells (5 × 10^6^ per mouse) in 150 μl of a 1:1 mixture of PBS and Matrigel (BD Biosciences) into the mammary fat pad. When the mean tumor volume reached approximately 100–120 mm^3^, the mice were randomly assigned to a treatment group and intraperitoneally (i.p.) injected with the TEDbody or a control Ab. If necessary, at 6 h after the TEDbody or control Ab injection, all mice were peritumorally injected with 5 × 10^6^ ex vivo-expanded CMVp-CTLs. After 24 h, the tumors were excised for immunohistochemistry (IHC) staining and an analysis of tumor-infiltrating CMVp-CTLs. IHC analysis of the tumor tissues excised from the mice was performed using the Zeiss LSM 710 system, as described before [14, 15]. The CMV-pMHCI complex was detected using DyLight 550-conjugated C1–17 Ab. Nuclei were stained with Hoechst 33342 for 5 min at 25 °C. After the tissue sections were washed three times with 0.1% Triton X-100 in PBS and mounted on slides with the Perma Fluor aqueous mounting medium, center-focused single z-section images were obtained on the Zeiss LSM 710 system. Quantitative analysis of the images was performed using ImageJ software (National Institutes of Health) [14].

For the analysis of tumor-infiltrating CMVp-CTLs, single-cell suspensions were prepared by mechanical dissociation of the tumors through a 70-mm wire-mesh screen. To determine the number of tumor-infiltrating CMVp-CTLs, the cells were counted using a hemocytometer, and we analyzed the prevalence of CMVp-CTLs labeled with a monoclonal Ab specific for CD8α and the PE-conjugated CMVp_495–503_-HLA-A*02:01 pentamer by flow cytometry. To identify the functional phenotype of CMVp-CTLs, the cells labeled with the monoclonal Ab specific for CD8α and the PE-conjugated CMVp_495–503_ peptide-HLA-A*02:01 pentamer were analyzed for T cell activation markers (CD69 and IFN-γ), as well as CD107a and granzyme B, for assessment of T cell cytotoxic function via flow cytometry. At least two hundred thousand events were collected using the FACSCalibur flow cytometer.

### In vivo antitumor experiments

For the MDA-MB-231 orthotopic xenograft tumor model, 4- to 6-week-old female NSG mice received an orthotopic injection of MDA-MB-231 cells (5 × 10^6^ cells per mouse) into the mammary fat pad. To set up the HCT116 xenograft tumor model, 4- to 6-week-old male NSG mice were inoculated subcutaneously, in the right thigh, with HCT116 cells (5 × 10^6^ per mouse). All cancer cells were injected in 150 μl of a 1:1 mixture of PBS and Matrigel (BD Biosciences). The appropriate number of mice per group (sample size) for comparison of multiple groups by one-way analysis of variance (ANOVA) was calculated according to the resource equation method using the following formula: *n* = (DF/k) + 1, where n = number of mice per group, DF = degrees of freedom with acceptable range between 10 and 20, and k = number of groups [34]. When the mean tumor volume reached approximately 100–120 mm^3^, the mice were randomly assigned to treatment groups, and the TEDbody or a control Ab, in addition to the IL-15/IL-15Rα-Fc protein (15 μg), was i.p. injected every 3 d in a dose/weight-matched manner (20 mpk). In a combination experiment, inCT†CMVp_480–516_ (20 mpk) combined with either the anti-PD1 Ab (5 mpk) or the anti-OX40 Ab (5 mpk) was i.p. injected, in addition to the IL-15/IL-15Rα-Fc protein (15 μg), every 3 d in a dose/weight-matched manner. At 6 h after the injection of the TEDbody, control Ab, or their combination, each mouse was injected in the tail vein with 10^7^ CMVp-CTLs (ex vivo-expanded cells derived from human PBMCs), every 6 d for the indicated period. Tumor volume (V) was evaluated using digital calipers and was estimated using the formula V = L × W^2^/2, where L and W are the long and short dimensions of a tumor, respectively [15]. Tumor growth inhibition (TGI) caused by the TEDbody, in comparison to TGI in the inCT group, was determined on day 3 after the last treatment according to the formula TGI (%) = [100 − (V_f_^TEDbody^ − V_i_^TEDbody^)/(V_f_^inCT^ – V_i_^inCT^) × 100], where V_i_ is the initial mean tumor volume in the TEDbody group or inCT treatment group and V_f_ is the final mean tumor volume in the TEDbody or inCT treatment group, as indicated by the superscript text [15]. If necessary, the tumors were excised on day 3, after the last treatment, for analyzing the CMV-pMHCI presentation (by IHC staining) and activation of tumor-infiltrating CMVp-CTLs, as described above. The mice were euthanized by CO_2_ asphyxiation, and some tumors were excised for histological analysis, as described in a previous study.

### Statistical analysis

Data are presented as a representative image for imaging experiments, mean ± SEM for pooled data, or mean ± SD for representative assays involving at least three independent experiments, unless specified otherwise. Differences between experimental groups and controls were analyzed for statistical significance by unpaired two-tailed Student’s *t*-test. One-way ANOVA with the Newman–Keuls multiple-comparison post hoc test was performed to determine the significance of in vivo tumor growth data using GraphPad Prism software (GraphPad, Inc.). No corrections were implemented in the statistical tests. *P* value < 0.05 was considered to denote statistical significance.

## Results

### Design and preparation of TEDbodies carrying various CMVp_495–503_-encompassing peptides

**Fig. 1:**
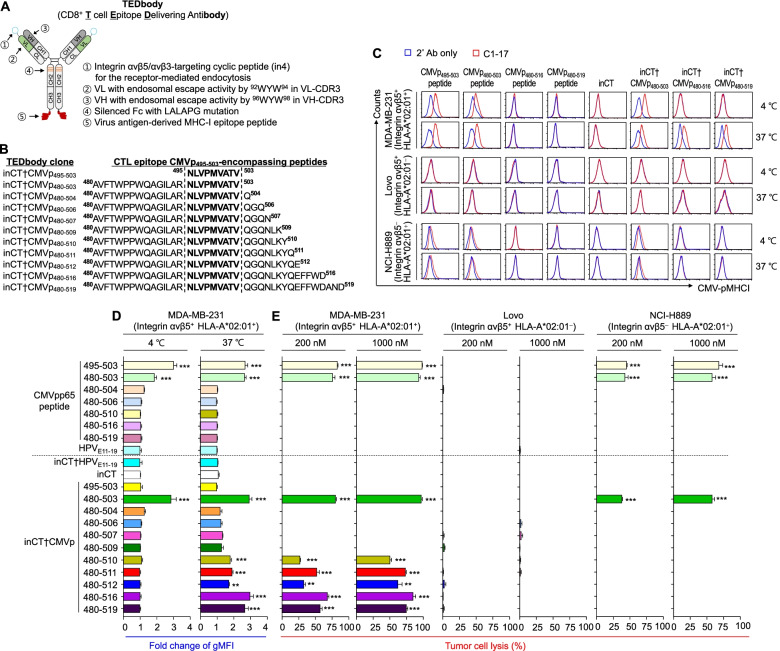
TEDbody-mediated CMV-pMHCI presentation drives efficient killing of target tumor cells by CMVp-CTLs. **A** Schematic of the TEDbody engineered for cytosolic delivery of an MHC-I-restricted viral CTL epitope peptide into the cytosol of target tumor cells. **B** Design of the TEDbodies carrying various CMVp_495–503_-encompassing peptides tested in this study and their nomenclature. A panel of CMVp_495–503_-encompassing peptides with N-terminally or N/C-extended sequences that were fused to the C-terminus of the heavy chain of inCT via an uncleavable 5-mer G_4_S linker. **C** A representative flow cytometric histogram of CMV-pMHCI display on the surface of cells as a consequence of extracellular treatment with the indicated synthetic peptide, TEDbody, or control Ab, as detected by the CMV-pMHCI-specific C1–17 Ab (red), compared to the control that used only the secondary Ab (blue). **D** Fold changes in the gMFI of CMV-pMHCI display, as determined by normalization to gMFI of the control involving only the secondary Ab. In (C) and (D), the indicated cells were treated with the indicated synthetic peptide, TEDbody, or control Ab (4 μM) at 4 °C for 3 h or at 37 °C for 18 h followed by flow cytometric analysis. All flow cytometric data for MDA-MB-231, LoVo, and NCI-H889 cells are shown in Fig. S3. In (D), bar graphs present the mean ± SEM (*n* ≥ 3). **P < 0.01 and ***P < 0.001 compared with HPV_E11–19_-treated cells for CMV pp65-derived peptides or compared with inCT-treated cells for the TEDbody/control Ab. **E** Percentage rates of tumor cell lysis by ex vivo-expanded CMVp-CTLs for integrin αvβ5^+^ HLA-A*02:01^+^ MDA-MB-231 cells, integrin αvβ5^+^ HLA-A*02:01^−^ LoVo cells, and integrin αvβ5^−^ HLA-A*02:01^+^ NCI-H889 cells, treated with the indicated synthetic peptide, TEDbody, or control Ab (0.2 or 1.0 μM) at 37 °C for 12 h prior to coculture with CMVp-CTLs for 18 h at an E:T ratio of 5:1. Percentage rates of lysis were determined by LDH quantification in the supernatant. The bar graphs present the mean ± SEM (*n* = 3)

For cytosolic delivery of viral CTL epitope peptides specifically into integrin αvβ5-expressing tumor cells, we used a tumor-specific cytotransmab, inCT, previously engineered to have two functional parts: (i) the light chain N-terminus-fused cyclic peptide (in4) specific to αvβ5 for receptor-mediated endocytosis, and (ii) VH and VL with an endosomal escape ability for relocation to the cytosol from an endosome [13, 14] (Fig. 1A). For the proof-of-concept experiment with a TEDbody, we chose to deliver the HLA-A*02:01-restricted CTL epitope of CMV pp65-derived CMVp_495–503_ into the cytosol of target tumor cells. To identify optimal fusion peptides for the TEDbody in terms of CMV-pMHCI presentation efficiency after cytosolic localization, we screened a panel of precursor peptides encompassing 9-mer mature epitope CMVp_495–503_ with N-terminally or N- and C(N/C)-terminally extended sequences by fusion with the C-terminus of the heavy chain of inCT via an uncleavable 5-mer G_4_S linker (Fig. 1B). To exclude nonspecific cellular uptake of the TEDbody by Fcγ receptor-expressing antigen-presenting cells, inCT with a silenced Fc domain carrying LALAPG mutations (L234A/L235A/P329G) was employed to abrogate the interactions with Fcγ receptors [35] (Fig. 1A). Hereafter, such TEDbody clones (i.e., CD8^+^ T cell epitope peptide-fused inCT) are referred to as “inCT†[peptide name]” (Fig. 1B). For example, the TEDbody carrying CMVp_495–503_ was named inCT†CMVp_495–503_. The designed TEDbodies were found to be well expressed in the correctly assembled form in a standard HEK293F transient expression system (Fig. S1).

### CMV-pMHCI presentation via a TEDbody carrying various CMVp_495–503_-encompassing peptides

Cell surface-expressed MHC-I molecules are often empty and permissive of direct loading at the cell surface with extracellularly provided epitope peptides [36]. To determine whether CMVp_495–503_-encompassing peptides were loaded onto HLA-A*02:01 at the cell surface without cellular internalization or after cellular uptake and processing, each synthetic peptide and TEDbody, either at 4 °C for 3 h or at 37 °C for 18 h, was extracellularly applied to human breast cancer MDA-MB-231 cells expressing both integrin αvβ5 and HLA-A*02:01 (Fig. S2). As controls, we used HLA-A*02:01^−^ integrin αvβ5^+^ human colorectal cancer LoVo cells and HLA-A*02:01^+^ integrin αvβ5^−^ human small cell lung cancer NCI-H889 cells (Fig. S2). At 4 °C, energy-dependent cellular internalization should not occur, and CMV-pMHCI formation results from extracellular surface loading. At 37 °C, the 18 h incubation was intended to enable cellular uptake and processing for the surface presentation of CMV-pMHCI. The cell surface presentation of CMV-pMHCI was monitored by flow cytometry using a high-affinity TCR-like Ab (C1–17) specific to CMV-pMHCI [27]. When pulsed with 9-mer CMVp_495–503_ or an N-extended 24-mer CMVp_480–503_ synthetic peptide, HLA-A*02:01^+^ MDA-MB-231 and NCI-H889 cells, but not HLA-A*02:01^−^ LoVo cells, were positively stained with the C1–17 Ab in proportion to the HLA-A*02:01 expression levels at both 4 °C and 37 °C (Fig. 1C, Fig. S2, and Fig. S3), indicating the direct extracellular surface loading of the peptides. On the contrary, C1–17 did not stain HLA-A*02:01^+^ cells pulsed with one of the N/C-extended peptides (CMVp_480–504_, CMVp_480–506_, CMVp_480–510_, CMVp_480–516_, CMVp_480–519_) or an off-target peptide, namely, HLA-A*02:01-restricted human papilloma virus (HPV)-16 E7 protein-derived 9-mer peptide, i.e., HPV_E11–19_ (^11^YMLDLQPETV^19^) [37] (Fig. 1C, D and Fig. S3). These results suggested that CMVp_495–503_-encompassing N-extended peptides can be extracellularly accommodated by HLA-A*02:01 if they have a correct C-terminus but cannot be extracellularly accommodated if they have extra residues at the C-terminus (even one amino acid residue), as seen with the ovalbumin (OVA)-derived ^257^SIINFEKL^264^ epitope binding to the murine H-2K^b^ MHC-I molecule [38].

For cells treated with a TEDbody, we observed that inCT†CMVp_480–503_-induced CMV-pMHCI formation on the surface of HLA-A*02:01^+^ cells at both 4 °C and 37 °C, but not on HLA-A*02:01^−^ cells, suggesting that CMVp_480–503_ in the Ab-fused form can be extracellularly loaded onto HLA-A*02:01 (Fig. 1C, D and Fig. S3). In contrast, inCT†CMVp_495–503_, carrying the mature 9-mer CMVp_495–503_ epitope with the N-terminal G_4_S linker residue, failed to form CMV-pMHCI even on HLA-A*02:01^+^ cells at both 4 °C and 37 °C, implying that the natural N-terminal flanking residues of CMVp_495–503_ are critical determinants of extracellular surface loading onto HLA-A*02:01, if they exist. Furthermore, the inability of inCT†CMVp_495–503_ to implement CMV-pMHCI display on the cell surface at 37 °C might be attributable to the absence of cellular proteolytic cleavage between the G_4_S linker and CMVp_495–503_ epitope. Additionally, TEDbodies carrying various N/C-extended peptides with relatively short C-terminal flanking sequence (i.e., CMVp_480–504_, CMVp_480–506_, CMVp_480–507_, and CMVp_480–509_) failed to cause CMV-pMHCI display on HLA-A*02:01^+^ cells at both 4 °C and 37 °C, in line with the inability of the N/C-extended peptides to attain extracellular surface loading onto HLA-A*02:01 (Fig. 1C, D). In contrast, TEDbodies carrying an N/C-extended peptide with longer C-terminal flanking sequences (CMVp_480–510_, CMVp_480–511_, CMVp_480–512_, CMVp_480–516_, or CMVp_480–519_) presented CMV-pMHCI on the surface of integrin αvβ5^+^ HLA-A*02:01^+^ cells only at 37 °C, but not at 4 °C, and did not on integrin αvβ5^+^ HLA-A*02:01^−^ cells at both 4 °C and 37 °C (Fig. 1C, D and Fig. S3). For integrin αvβ5^−^ HLA-A*02:01^+^ NCI-H889 cells (Fig. S2), the CMV-pMHCI presentation was detectable only with extracellular surface loading-capable inCT†CMVp_480–503_ but not with N/C-extended peptide-carrying TEDbodies (Fig. 1C). The above findings meant that the N/C-extended peptides fused to inCT cannot be extracellularly loaded onto HLA-A*02:01, as seen with the N/C-extended peptides themselves. Nonetheless, they can be displayed in the form of CMV-pMHCI on the cell surface by TEDbody-mediated cellular delivery and processing if they have a long enough C-terminal flanking sequence (i.e., at least beyond the residue number 510, as is the case with inCT†CMVp_480–510_) to be recognized and cleaved by cytosolic proteasomes [39]. Overall, the above data indicated that a TEDbody can intracellularly deliver N/C-extended CMVp_495–503_-encompassing peptides to be displayed in the pMHCI form of CMV-pMHCI on the surface of target cells expressing both integrin αvβ5 and HLA-A*02:01.

### Ex vivo-expanded CMVp-CTLs lyse CMV-pMHCI-presenting tumor cells

To prepare CMV-pMHCI-specific CTLs, i.e., CMVp-CTLs, we first screened PBMCs from healthy individuals (*n* = 75) and found that 62% of the tested individuals (28 out of 45) among HLA-A*02:01^+^ individuals (45 out of 75) had circulating CMVp-CTLs (Table S3). The prevalence of CMVp-CTLs ranged from 0.14 to 5.34% of all CD8^+^ T cells (median, 1%) from CMV-seropositive PBMCs (Fig. S4 and Table S3), in line with previous reports [9, 23]. For use in in vitro and in vivo experiments, PBMCs carrying CMVp-CTLs from 12 donors were chosen for stimulation for 14 to 18 d with the CMVp_495–503_ peptide and cytokines to expand CMVp-CTLs to the range of 4.47 to 97.5% among all CD8^+^ T cells (median, 60%; Table S4). Memory phenotype analysis of CMVp-CTLs by means of surface expression of CCR7 and CD45RA [40] revealed that the majority of prestimulated CMVp-CTLs represented a CCR7^−^ CD45RA^−^ effector memory (T_EM_) subset and a CCR7^−^ CD45RA^+^ terminally differentiated effector memory (T_EMRA_) subset, but poststimulated CMVp-CTLs predominantly represented the T_EM_ subset (Fig. S5A and Table S4), which was consistent with other reports [9, 23]. Thus, the ex vivo-expanded CMVp-CTLs had the capacity to be rapidly activated and to be cytotoxic to CMV-pMHCI-presenting cells. At an effector-to-target cell ratio (E:T) of 5:1, the CMVp-CTLs efficiently lysed HLA-A*02:01^+^ cells pulsed with extracellular surface loading-capable peptides (CMVp_495–503_ or CMVp_480–503_) but not those incapable N/C-extended peptides, and there was no lysis of off-target peptide (HPV_E11–19_)-pulsed HLA-A*02:01^+^ cells or CMVp_495–503_-pulsed HLA-A*02:01^−^ cells (Fig. 1E). These findings indicated a CMV-pMHCI-dependent killing ability and specificity of the ex vivo-expanded CMVp-CTLs.

### CMVp-CTLs recognize and kill tumor cells presenting CMV-pMHCI via a TEDbody

To test whether TEDbody-mediated CMV-pMHCI presentation renders the marked tumor cells susceptible to lysis by CMVp-CTLs, TEDbodies were incubated with tumor cells for 12 h to ensure internalization and cellular processing for CMV-pMHCI presentation, followed by coculturing for another 18 h with ex vivo-expanded CMVp-CTLs, at an E:T ratio of 5:1. Compared with the control inCT, TEDbodies that had shown the ability to implement CMV-pMHCI presentation at 37 °C substantially induced the lysis of HLA-A*02:01^+^ MDA-MB-231 cells, but not HLA-A*02^−^ LoVo cells, in a concentration-dependent manner (Fig. 1E). This evidence suggested that TEDbody-mediated CMV-pMHCI presentation can activate CMVp-CTLs among PBMCs to elicit immune responses. The cell lysis potency of the TEDbodies roughly correlated with the magnitude of CMV-pMHCI surface display at 37 °C. In contrast, the TEDbodies carrying either off-target HPV_E11–19_ or a CMV-pMHCI display-incapable peptide only negligibly triggered cell lysis. Notably, the TEDbody carrying a N/C-extended CMVp_480–516_ or CMVp_480–519_ peptide that requires cellular uptake to present CMV-pMHCI manifested the strongest ability to induce tumor cell lysis; the lysis magnitude was comparable to that induced by CMVp_495–503_ peptide pulsing (Fig. 1E). In integrin αvβ5^−^ HLA-A*02:01^+^ NCI-H889 cells, the lysis was caused by extracellular surface loading-competent inCT†CMVp_480–503_, but not by the N/C-extended peptide-bearing TEDbodies requiring cellular uptake for CMV-pMHCI presentation (Fig. 1E). These results demonstrated the target cell specificity of the cellular internalization-requiring TEDbody, specifically, its ability to drive CMV-pMHCI presentation on the surface of cells expressing both integrin αvβ5 and HLA-A*02:01 to be recognized and lysed by CMVp-CTLs.

### TEDbody-mediated CMV-pMHCI presentation proceeds via the conventional MHC-I antigen-processing pathway

**Fig. 2:**
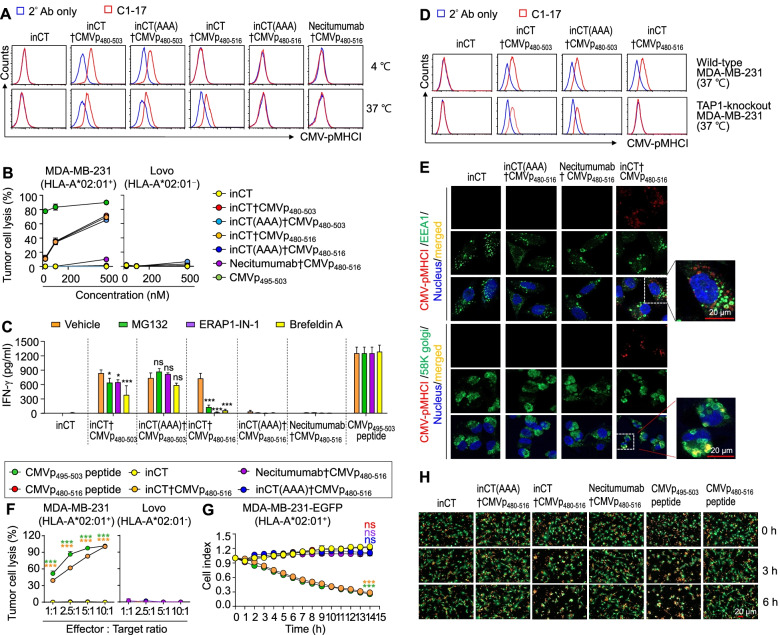
TEDbody-mediated CMV-pMHCI presentation proceeds via the conventional MHC-I antigen-processing pathway. **A** A representative flow cytometric histogram of CMV-pMHCI display on the surface of MDA-MB-231 cells, detected by the CMV-pMHCI-specific C1–17 Ab (red) in comparison with the control involving only the secondary Ab (blue). The cells were treated with the indicated peptide, TEDbody, or control Ab (4 μM) at 4 °C for 3 h or at 37 °C for 18 h prior to flow cytometric analysis. **B** Percentage rates of tumor cell lysis by ex vivo-expanded CMVp-CTLs after the cancer cells were treated with the indicated peptide, TEDbody, or control Ab (20, 100, or 500 nM) for 12 h at 37 °C, prior to coculture with CMVp-CTLs for 18 h at an E:T ratio of 5:1. **C** IFN-γ secretion caused by the activation of CMVp-CTLs in response to CMV-pMHCI presentation on MDA-MB-231 cells after the cells were treated with the indicated peptide, TEDbody, or control Ab (0.2 μM) for 12 h at 37 °C in the absence or presence of MG132 (20 μM), ERAP1-IN-1 (20 μM), or brefeldin A (200 nM). The bar graphs show the mean ± SEM (*n* ≥ 3). **D** A representative flow cytometric histogram of CMV-pMHCI display on the surface of wild-type and TAP1 knockout MDA-MB-231 cells (red), compared to the control involving only the secondary Ab (blue). The cells were treated with the indicated TEDbody or control Ab (4 μM) at 37 °C for 18 h prior to flow cytometric analysis with the C1–17 Ab. **E** Representative confocal fluorescence microscopy images of MDA-MB-231 cells treated with the indicated TEDbody or control Ab (4 μM) at 37 °C for 18 h and monitoring of colocalization of CMV-pMHCI (red) with early endosome marker EEA1 (green) or a Golgi marker called 58 K Golgi (green). Nuclei were stained with Hoechst 33342 (blue). Scale bar: 20 μm. The images are representative of three independent experiments. **F** Percentage rates of tumor cell lysis by ex vivo-expanded CMVp-CTLs, after the cancer cells were treated with the indicated peptide, TEDbody, or control Ab (1 μM) at 37 °C for 12 h, prior to coculture with CMVp-CTLs for 18 h at the indicated E:T ratio. **G** and **H** Real-time kinetics of TEDbody-induced cell lysis of MDA-MB-231-EGFP cells by ex vivo-expanded PKH26-labeled CMVp-CTLs (G) and representative time-lapse fluorescence microscopy images (H). MDA-MB-231-EGFP cells treated with the indicated peptide, TEDbody, or control Ab (1 μM) for 12 h and then cocultivated with PKH26-labeled CMVp-CTLs at an E:T ratio of 3:1 inside the Lionheart FX automated microscopy system for the indicated periods. Lysis of MDA-MB-231-EGFP cells (green) by PKH26-labeled CMVp-CTLs (red) was registered based on a loss of the EGFP signal. In (G), the cell index refers to green fluorescence intensity from the total cancer cell area after normalization to that from the total cancer cell area at time point 0. In (H), scale bar: 20 μm. In (B), (C), (F), and (G), error bars present the mean ± SEM (*n* = 3). In (B), (C), (F), and (G), *P < 0.05, **P < 0.01, and ***P < 0.001 compared with the vehicle-treated control (B,C) or inCT-treated control (F,G); ns: not significant

To further dissect the TEDbody-mediated CMV-pMHCI presentation, we generated peptide-fused Ab controls via fusion of the N-extended CMVp_480–503_ or N/C-extended CMVp_480–516_ to endosomal escape-incapable Abs [anti-EGFR therapeutic Ab called necitumumab (Portrazza™) or inCT (AAA) [13, 14]] in the same manner as with a TEDbody. Although the anti-EGFR Ab and endosomal escape motif-deficient inCT (AAA) get internalized into the cells through a specific receptor of EGFR or integrin αvβ5, respectively, they are not expected to deliver the fused peptide into the cytosol of target cells because of the absence of the endosomal escape ability. Both inCT†CMVp_480–503_- and inCT (AAA)†CMVp_480–503_-induced CMV-pMHCI presentation at both 4 °C and 37 °C and elicited cytolysis by CMVp-CTLs at 37 °C in an HLA-A*02:01-restricted manner (Fig. 2A,B), thereby confirming the extracellular surface loading capability of the CMVp_480–503_ peptide even in the Ab-fused form. On the contrary, CMVp_480–516_-fused necitumumab and inCT (AAA) failed to implement CMV-pMHCI presentation at 4 °C and 37 °C and did not induce cytolysis by CMVp-CTLs, in contrast to the efficient CMV-pMHCI presentation and cytolysis of inCT†CMVp_480–516_-treated HLA-A*02:01^+^ cells in proportion to the concentration (Fig. 2A,B). These findings indicate that TEDbody-mediated cytosolic delivery of N/C-extended CMVp_480–516_ is essential for surface presentation of CMV-pMHCI.

To determine the involvement of intracellular processing in TEDbody-induced CMV-pMHCI presentation, we examined effects of a proteasome inhibitor (MG132), an ER-resident aminopeptidase 1 inhibitor (ERAP1-IN-1) [41], and an inhibitor of vesicle-mediated transport from the ER to the Golgi apparatus (brefeldin A) [42] on the activation of CMVp-CTLs mediated by a TEDbody through CMV-pMHCI display. CMVp-CTL activation was detected by quantifying IFN-γ released into the supernatant to exclude any possible cytotoxic impact of the inhibitor on tumor cells. The three inhibitors did not significantly affect the CMVp-CTL activation induced by pulsing with either the CMVp_495–503_ peptide or inCT (AAA)†CMVp_480–503_ (Fig. 2C), confirming their extracellular surface loading ability without the need for further intracellular processing. On the other hand, the activation of CMVp-CTLs by inCT†CMVp_480–503_ was significantly inhibited (but was still substantial) by treatment with each of the three inhibitors (Fig. 2C). These findings indicated that inCT†CMVp_480–503_-mediated CMV-pMHCI formation proceeds via two pathways: i) extracellular surface loading onto HLA-A*02:01 at the cell surface, as observed at 4 °C (Fig. 2A), and ii) conventional class I antigen processing after cytosolic access. In contrast, inCT†CMVp_480–516_-mediated activation of CMVp-CTLs was almost completely abrogated by the presence of each of the three inhibitors (Fig. 2C). One study elucidated that cytosolically generated CMVp_495–503_-encompassing peptides are efficiently transported into the ER by TAP [39]. A knockout of TAP1 in MDA-MB-231 cells (Fig. S6A, B) substantially reduced the magnitude of the CMV-pMHCI surface presentation caused by inCT†CMVp_480–516_ but only slightly by inCT†CMVp_480–503_ or inCT (AAA)†CMVp_480–503_ (Fig. 2D), indicating that TAP1 is involved in the transport of cytosolically processed CMVp_480–516_-derived epitope precursor peptides from the cytosol into the ER.

Next, we visualized the intracellular trafficking of CMV-pMHCI by its costaining with EEA1 (specific for early endosomes) or with 58 K Golgi (specific for the Golgi apparatus) [33]. In the controls of endosomal escape motif-deficient necitumumab and inCT (AAA) carrying CMVp_480–516_, CMV-pMHCI was not detectable in any cellular compartment (Fig. 2E), which was consistent with the inability to cause CMV-pMHCI display. However, in cells treated with inCT†CMVp_480–516_, CMV-pMHCI was present inside the cells and colocalized with 58 K Golgi but not with EEA1 (Fig. 2E), thus pointing to the trafficking of CMV-pMHCI through the ER–Golgi pathway for surface presentation. Collectively, the above data suggested that inCT†CMVp_480–516_-induced CMV-pMHCI presentation proceeds entirely through the class I antigen-processing pathway [12], as follows: i) cytosolic delivery of CMVp_480–516_ and its cleavage by a cytosolic proteasome to generate precursor peptides with the correct C-terminus; ii) transport of the precursors into the ER by TAP and trimming of the residues of the N-extended sequence (if any) by ERAPs in the ER to generate the mature epitope (CMVp_495–503_) for loading onto HLA-A*02:01 to form CMV-pMHCI; and iii) exocytosis of CMV-pMHCI through the ER–Golgi transport pathway for surface presentation, as in the natural processing of the CMV pp65 antigen [39].

### TEDbody-induced tumor cell lysis by CMVp-CTLs is proportional to the number of effector cells and incubation time

By means of inCT†CMVp_480–516_ requiring cytosolic processing for CMV-pMHCI presentation, we further characterized the lysis of target tumor cells by CMVp-CTLs. The magnitude of tumor cell lysis driven by inCT†CMVp_480–516_ increased in proportion to the E:T ratio only for HLA-A*02:01^+^ tumor cells but not for HLA-A*02:01^−^ tumor cells (Fig. 2F). For a real-time cell lysis assay, we treated MDA-MB-231-EGFP cells stably expressing EGFP (Fig. S6C) with inCT†CMVp_480–516_ for 12 h and then cocultured them with ex vivo-expanded CMVp-CTLs labeled with red fluorescent dye PKH26, while monitoring a decrease in fluorescence intensity of EGFP as an indicator of MDA-MB-231-EGFP cell lysis. In kinetic experiments, the treatment of inCT†CMVp_480–516_ led to rapid lysis of MDA-MB-231-EGFP cells (Fig. 2G, H), i.e., caused more than 70% lysis after 14 h, similar to that of CMVp_495–503_ peptide. In contrast, cytosolic access-incapable inCT (AAA)†CMVp_480–516_ and necitumumab†CMVp_480–516_ as well as the extracellular surface loading-incapable N/C-extended CMVp_480–516_ peptide triggered negligible cell-killing activity (Fig. 2G,H). These results clearly indicated that TEDbody-mediated cytosolic delivery of CMVp_480–516_ resulted in efficient presentation of CMV-pMHCI on the surface of target tumor cells for their lysis by CMVp-CTLs.

### TEDbody-mediated CMV-pMHCI presentation activates CMVp-CTLs in vivo

**Fig. 3:**
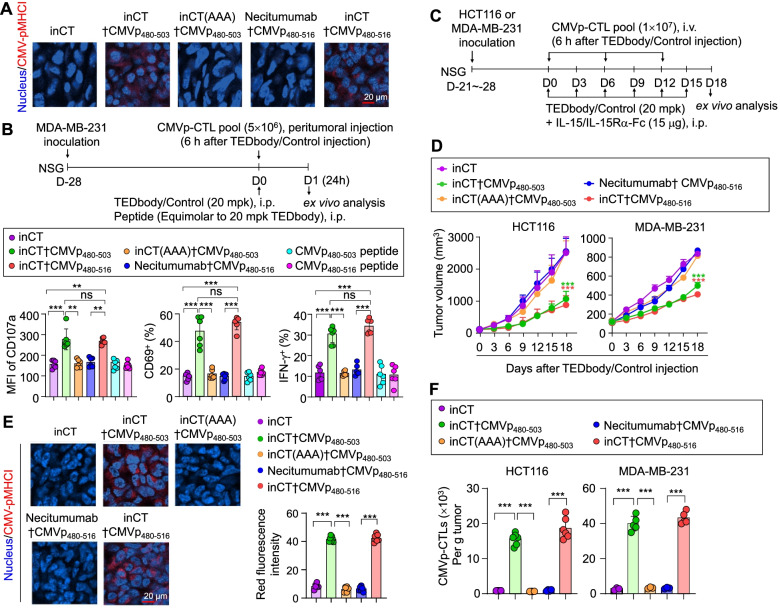
TEDbody-mediated in vivo CMV-pMHCI presentation suppresses tumor growth by redirecting adoptively transferred CMVp-CTLs to tumor cells in mice. **A** IHC detection of CMV-pMHCI (red) on MDA-MB-231 tumor tissues excised from NSG mice bearing a preestablished MDA-MB-231 cell-derived tumor xenograft (100–120 mm^3^), 24 h after a single i.p. injection of the indicated TEDbody or control Ab (20 mpk). Images are representative of three independent experiments; additional images are shown in Fig. S7A. Nuclei were stained with Hoechst 33342 (blue). Scale bar: 20 μm. **B** Functional phenotype analysis of CMVp-CTLs from MDA-MB-231 tumor tissues, excised from NSG mice bearing a preestablished MDA-MB-231 tumor (100–120 mm^3^), 24 h after a single i.p. injection of the indicated TEDbody, control Ab (20 mpk), or peptide (equivalent molar amount of 20 mpk TEDbody), followed 6 h later by peritumoral injection of ex vivo-expanded CMVp-CTLs (5 × 10^6^ cells), as described in the upper panel. Each symbol represents a value obtained from an individual mouse. The data from inCT and inCTp_480–503_ were pooled from two independent experiments with at least three mice per group. The bar graphs present the mean ± SEM (*n* ≥ 3). **C** The treatment scheme for assessing in vivo antitumor efficacy of the TEDbody or a control Ab in conjunction with adoptive transfer of ex vivo-expanded CMVp-CTLs in NSG mice carrying a preestablished HCT116 cell-derived subcutaneous tumor xenograft or MDA-MB-231 cell-derived orthotopic tumor xenograft (100–120 mm^3^). The arrows indicate each time point for the treatment or assay. **D** Tumor growth, measured as the average tumor volume, in response to the indicated treatment, as described in (C). Error bars: ±SEM (*n* = 9 to 14 per group for HCT116 tumors, *n* = 8 to 13 per group for MDA-MB-231 tumors). Data were pooled from two independent experiments with at least four mice per group. **E** and **F** IHC detection of CMV-pMHCI (red) on tumor tissues (E) and the number of tumor-infiltrating CMVp-CTLs per gram of a tumor (F) excised from mice on day 3 after the last treatment, as described in (C). In (E), nuclei were stained with Hoechst 33342 (blue), and images are representative of three independent experiments; additional images are shown in Fig. S7B. Scale bar: 20 μm. The right panel shows the quantification of red fluorescence intensity, obtained by ImageJ software. Error bars, ±SD of 2 fields per tumor (*n* = 3 per group). In (F), bar graphs present the mean ± SEM (n ≥ 3 different tumors). In (B), (D), and (F), **P < 0.01 and ***P < 0.001 denote a significant difference between the indicated groups (B and F) or a significant difference from the inCT group (B, D, and F), as determined by one-way ANOVA with the Newman–Keuls post hoc test; ns: not significant

To determine whether a TEDbody can label tumor cells with CMV-pMHCI in vivo, a test TEDbody at 20 mg/kg (mpk) was i.p. injected once into immunodeficient NSG mice bearing preestablished orthotopic MDA-MB-231 breast cancer cell-derived xenografts having an average tumor volume of 100–120 mm^3^. At 24 h postinjection, the tumors were excised and subjected to IHC analysis of the surface expression of CMV-pMHCI. Notably, treatment with either inCT†CMVp_480–503_ or inCT†CMVp_480–516_ yielded CMV-pMHCI presentation of similar magnitudes near the plasma membrane and inside the tumor cells (Fig. 3A and Fig. S7A), thereby pointing to in vivo capacity for inducing CMV-pMHCI presentation on tumor cells after systemic administration. In contrast, neither inCT (AAA)†CMVp_480–503_ nor necitumumab†CMVp_480–516_ had this effect. Although inCT (AAA)†CMVp_480–503_ caused CMV-pMHCI display in vitro via extracellular surface loading, it failed in vivo, in line with some reports that pMHCI formation through extracellular surface loading rarely occurs in vivo [11, 36]. Consequently, the in vivo presentation of CMV-pMHCI with the help of inCT†CMVp_480–503_ seemed to be mediated mainly by the intracellular antigen-processing pathway, as was the case for inCT†CMVp_480–516_.

To test whether the TEDbody-mediated CMV-pMHCI presentation can activate CMVp-CTLs in the TME, we repeated the in vivo experiment with the i.p. injection of the TEDbody (20 mpk), and 6 h later, peritumorally injected ex vivo-expanded CMVp-CTLs (Fig. 3B). At 24 h later, the tumors were excised for functional phenotype analysis of tumor-infiltrating CMVp-CTLs by fluorescently activated cell sorting. Compared with the controls [CMVp_480–503_ peptide, CMVp_480–516_ peptide, inCT, inCT (AAA)†CMVp_480–503_, or necitumumab†CMVp_480–516_], treatment with either inCT†CMVp_480–503_ or inCT†CMVp_480–516_ upregulated CD107, a marker of degranulation activity [9], on the surface of CMVp-CTLs (Fig. 3B), indicating an augmented cytotoxic ability in response to CMV-pMHCI. Consistent with this, the prevalence of CD69- and IFN-γ-producing CMVp-CTLs among the tumor-infiltrating cells was substantially higher after treatment with either inCT†CMVp_480–503_ or inCT†CMVp_480–516_ than after treatment with the control substances (Fig. 3B). These results meant that TEDbody-mediated CMV-pMHCI presentation serves as antigenic stimulation in the TME to augment the activation and cytotoxic properties of tumor-infiltrating CMVp-CTLs.

### In vivo antitumor efficacy of the TEDbody in immunodeficient mice bearing human tumors

To assess the in vivo antitumor activity of the TEDbody in conjunction with adoptive transfer of ex vivo-expanded CMVp-CTLs, we treated NSG mice bearing preestablished orthotopic MDA-MB-231 xenografts or subcutaneous HCT116 colorectal cancer cell-derived xenografts having an average tumor volume of 100–120 mm^3^ with i.p. injection of the TEDbody or one of control substances (20 mpk) plus IL-15/IL-15Rα-Fc (15 μg); 6 h later, we intravenously injected ex vivo-expanded CMVp-CTLs (10^7^ cells; Fig. 3C). The TEDbody was injected every 3 d for a total of six doses, and CMVp-CTLs were administered every 6 d for a total of three doses (Fig. 3C). The IL-15/IL-15Rα-Fc protein, known as P22339 [30], was concomitantly injected to increase the survival of the transferred CMVp-CTLs. Compared with the inCT-treated control, cytosolic access-incapable inCT (AAA)†CMVp_480–503_ and necitumumab†CMVp_480–516_ failed to inhibit tumor growth (Fig. 3D), which was consistent with their in vivo inability to cause CMV-pMHCI presentation (Fig. 3A). By contrast, inCT†CMVp_480–516_ and inCT†CMVp_480–503_ markedly slowed the tumor growth, manifesting an in vivo antitumor activity via the transferred CMVp-CTLs (Fig. 3D and Fig. S8). Compared to the inCT-treated control, they showed similar antitumor potency levels, with TGI (at the end of treatment) of 46% (inCT†CMVp_480–503_) and 58% (inCT†CMVp_480–516_) for MDA-MB-231 xenografts and 61% (inCT†CMVp_480–503_) and 68% (inCT†CMVp_480–516_) for HCT116 xenografts. When the tumors were excised on day 3 after the last treatment and analyzed via IHC staining, CMV-pMHCI presentation was detectable in the tumors treated with inCT†CMVp_480–516_ or inCT†CMVp_480–503_ but not in those treated with the controls (Fig. 3E and Fig. S7B). Moreover, compared to the control groups, treatment with CMV-pMHCI-presenting TEDbody increased the number of tumor-infiltrating CMVp-CTLs by ~ 15-fold (Fig. 3F), indicating that TEDbody-mediated CMV-pMHCI presentation induced efficient infiltration of the transferred CMVp-CTLs into tumor tissue and/or stimulated their proliferation in the TME. Collectively, the above results explained the in vivo antitumor mechanisms of action of TEDbody, namely, the marking of target tumor cells with CMV-pMHCI for recognition and killing by transferred CMVp-CTLs.

### Combination of the TEDbody with an anti-OX40 agonistic ab enhances the antitumor activity

**Fig. 4:**
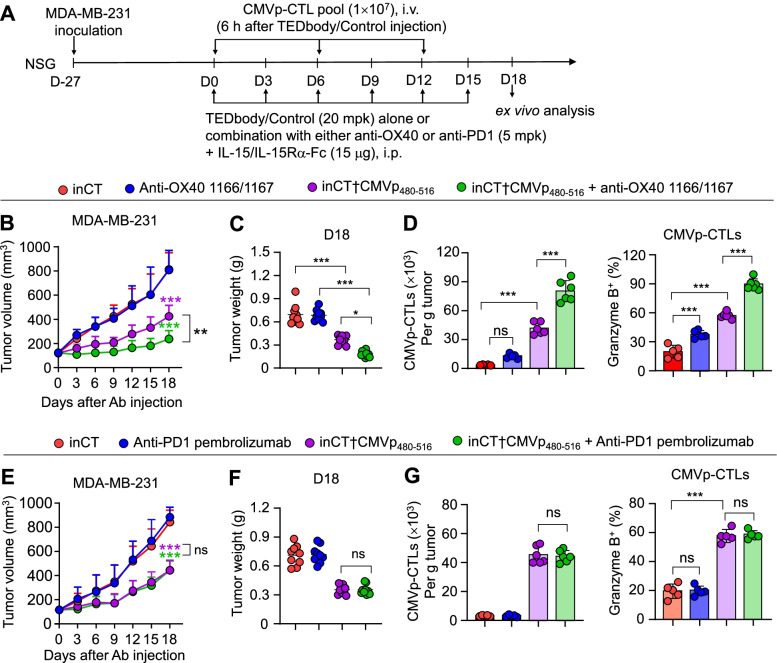
Combination of TEDbody with anti-OX40 agonistic Ab augments antitumor activity. **A** The treatment scheme for evaluating the effect of combination of either the TEDbody or a control Ab with either the anti-OX40 agonistic 1166/1167 Ab (anti-OX40) or the anti-PD1 antagonistic Ab (pembrolizumab: anti-PD1 in the figure) in conjunction with adoptive transfer of ex vivo-expanded CMVp-CTLs in NSG mice carrying a preestablished MDA-MB-231 orthotopic tumor xenograft (100–120 mm^3^). The arrows indicate each time point for a treatment or assay. **B** and **E** Tumor growth, measured as the average tumor volume, in response to the indicated treatment, as described in (A). Error bars: ±SEM (*n* = 9 to 11 per group). Data were pooled from two independent experiments with at least with four mice per group. **C**, **D**, **F** and **G** Tumor weight (C and F) and the number of tumor-infiltrating CMVp-CTLs per gram of a tumor and percentages of granzyme B-expressing tumor-infiltrating CMVp-CTLs (D and G), determined on day 3 after the last administration of the indicated treatment, as described in (A). Each symbol represents a value for one tumor from an individual mouse; midlines indicate the mean values. In (D) and (G), error bars denote ±SEM. In (B) to (G), *P < 0.05, **P < 0.01, and ***P < 0.001 indicate a significant difference between the indicated groups or a significant difference from the inCT group (B and E), determined by one-way ANOVA with the Newman–Keuls post hoc test; ns: not significant

In studies on adoptive transfer of CTLs for cancer immunotherapy, a T cell costimulatory agonist or inhibitory antagonist has been added to enhance the antitumor effects [9, 11]. Because a T cell costimulatory molecule called OX40 was found to be upregulated in the ex vivo-expanded effector memory CMVp-CTLs (Fig. S5B, C), we conducted an in vivo assay by combining inCT†CMVp_480–516_ with an anti-OX40 1166/1167 agonistic Ab (anti-OX40; Fig. S9) [28] in NSG mice with preestablished MDA-MB-231 xenografted tumors (Fig. 4A). Compared with the inCT-treated control, the combined treatment with inCT†CMVp_480–516_ and anti-OX40 markedly enhanced the in vivo antitumor activity, with a TGI of 83% which was notably higher than that of the monotherapy with either inCT†CMVp_480–516_ (56%) or anti-OX40 (0%; Fig. 4B,C and Fig. S10). Compared with each monotherapy, the combined treatment further increased the number of tumor-infiltrating CMVp-CTLs and potentiated the cytotoxic effector function, as evidenced by expression analysis of granzyme B (Fig. 4D). A portion of CMVp-CTLs expressed inhibitory receptors, such as PD1, LAG-3, and/or TIGIT (Fig. S5B, C). To explore any blocking effects of PD1, the representative inhibitory receptor for CTL exhaustion, on the antitumor activity of CMVp-CTLs, we combined inCT†CMVp_480–516_ with an anti-PD1 antagonistic Ab, pembrolizumab (Fig. S9). Compared to treatment with inCT†CMVp_480–516_ alone, the combined treatment did not provide any additional antitumor effects (Fig. 4E,F) nor did it increase the number and cytotoxicity of tumor-infiltrating CMVp-CTLs (Fig. 4G), even though PD1 and its ligand PD-L1 were expressed on the surface of CMV-CTLs (Fig. S5C) and MDA-MB-231 cells (Fig. S2), respectively. This suggested that a combination of a TEDbody with an anti-OX40 agonist, but not an anti-PD1 antagonist, is a viable approach to enhancing the proliferation and cytotoxic effector function of transferred CMVp-CTLs in the TME.

## Discussion

**Fig. 5:**
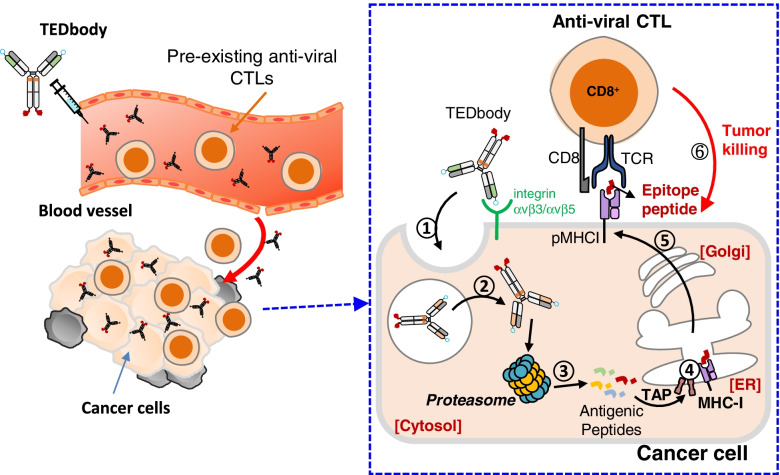
A schematic diagram of the proposed mode of action of a TEDbody in cancer immunotherapy. A TEDbody delivers a fused MHC-I-restricted viral CTL epitope peptide into the cytosol of integrin αvβ5-expressing cancer cells to be processed for surface presentation by cognate MHC-I, thereby rendering the marked cancer cells recognizable and killable by pre-existing antiviral CTLs arising from common human viral infections in cancer patients as follows: 1) binding to the tumor-associated receptor, integrin αvβ5, for cellular internalization; 2) cytosolic localization through endosomal escape; 3) proteasomal cleavage and degradation generating CTL epitope precursor peptides; 4) ER uptake by TAP and N-terminal trimming of the precursor peptides to generate the mature CTL epitope, followed by its binding to cognate MHC-I; 5) cell surface presentation of the pMHCI through the ER-to-Golgi pathway; 6) recognition and lysis of pMHCI-presenting cells by pre-existing pMHCI-specific antiviral CTLs

Cytosolic delivery of an MHC-I antigen into target tumor cells that lack tumor antigens amenable to immunotherapy remains a serious limitation but holds great potential as a gateway to the development of a therapeutic cancer vaccine, if achievable. Our study revealed an ability of a TEDbody to deliver an MHC-I-restricted viral CTL epitope peptide into the cytosol of integrin αvβ5-expressing target tumor cells for pMHCI presentation on the cell surface, which is a prerequisite step for priming the target cells for recognition and lysis by pre-existing antiviral CTLs arising from common viral infections in cancer patients (Fig. 5). To simulate tumor cells infected by human CMV, we generated TEDbodies carrying various CMVp_495–503_-encompassing peptides for surface expression of CMV-pMHCI via the conventional MHC-I antigen-processing pathway. The TEDbody-mediated CMV-pMHCI presentation redirected CMVp-CTLs of CMV-seropositive donors to recognize and kill target tumor cells in vitro and suppressed tumor growth in immunodeficient mouse models. Therefore, the TEDbody is a useful technology for cytosolic delivery of MHC-I-restricted viral peptides mimicking the natural presentation pathway of an MHC-I viral antigen, and therefore, may lead to possible therapeutic cancer vaccines directly targeting tumor cells rather than antigen-presenting cells.

Several approaches have been explored to deliver viral CTL epitope peptides to tumor cells with the aim of simulating cancer cells that are virally infected, with consequent redirection of the corresponding antiviral CTLs to the tumor. The previous approaches can be classified into two: (i) extracellular surface loading without cellular uptake [6, 8–10], and (ii) endosomal loading after cellular internalization [11]. As for the first approach of extracellular surface loading, intratumoral injection of various synthetic viral epitope peptides, including CMVp_495–503_ peptide itself, has been investigated but has resulted in very weak in vivo antitumor activity as monotherapy [6]. Another extracellular surface loading strategy is to take advantage of a tumor-targeting Ab–peptide epitope conjugate [9, 10] in which a mature peptide epitope is conjugated with an Ab via a linker cleavable by a tumor-associated protease such that the peptide epitope is proteolytically released onto the tumor cell surface and then extracellularly loaded onto MHC-I to form pMHCI without cellular uptake. The Ab–peptide epitope conjugates that were designed to release CMVp_495–503_ coated tumor cells with CMV-pMHCI for recognition by CMVp-CTLs, resulting in delayed tumor growth in mouse models, particularly when combined with immune checkpoint-blocking Abs [9]. However, in our study, we noted extracellular surface loading of N-extended CMVp_480–503_ (even in the form of fusion to an IgG Ab via an uncleavable G_4_S linker) onto HLA-A*02:01 with the formation of functional CMV-pMHCI reactive with CMVp-CTLs, suggesting that proteolytic cleavage of Ab-fused CTL epitopes at the cell surface is not a prerequisite for MHC-I loading. As for the second approach involving endosomal loading after cellular internalization, Ab-targeted pathogen-derived peptides have been generated by conjugation of an Epstein–Barr virus-derived CTL epitope peptide to a tumor-targeting Ab via a cleavable (reducible) disulfide bond in the endosomal environment after cellular endocytosis [11]. However, the necessity of the presence of a Cys residue in the CTL epitope sequence may limit the applicability of Ab-targeted pathogen-derived peptide technology. In contrast to TEDbody, the previous two approaches do not require cytosolic access and processing for cell surface presentation of a CTL epitope peptide on MHC-I. As demonstrated in studies with a pharmacological inhibitor and TAP1 knockout MDA-MB-231 cells treated with inCT†CMVp_480–516_, a TEDbody delivers MHC-I-restricted peptides into the cytosol of target tumor cells for pMHCI presentation through the endogenous MHC-I antigen presentation pathway [12, 39]. Although the surface loading of inCT (AAA)†CMVp_480–503_ onto HLA-A*02:01 to form CMV-pMHCI gave rise to significant cytolytic activity of CMVp-CTLs in vitro in our study, the in vivo CMV-pMHCI presentation and antitumor activity were negligible, suggesting that extracellular surface loading does not take place efficiently in vivo, as demonstrated in previous studies [6, 11, 36].

For the formation of the pMHCI complex, MHC-I molecules bind short peptides that are typically 8–11 amino acid residues in length (with a 9-mer preferred) with both ends tucked inside the binding groove of MHC-I [36]. In the current study, following the size restriction of MHC-I, the N/C-extended peptides and their fused form in a TEDbody failed to induce CMV-pMHCI via extracellular pulsing. In contrast, extracellular pulsing of N-extended peptide CMVp_480–503_ with the correct C-terminus in either the free or Ab-fused form in a TEDbody caused extracellular loading onto HLA-A*02:01 at 4 °C, thereby generating CMV-pMHCI. Moreover, the N-extended peptide-loaded CMV-pMHCI can also be recognized by CMVp-CTLs, as evidenced by the cytolytic activity at 37 °C. There are some documented cases of unconventional presentation of either N- or C-terminally extended peptides (but never peptides that are N/C-extended at the same time) on a cognate MHC-I molecule, including HLA-A*02:01, via structural changes either within the MHC-I that opens the confined binding groove (“protrusion”) or within the peptide itself (“bulging”) [43]. Nonetheless, unlike inCT†CMVp_480–503_, inCT†CMVp_495–503_ with the N-terminal artificial G_4_S linker sequence failed to be loaded onto HLA-A*02:01 in our study, indicating that N-terminal endogenous sequences flanking the matured epitope strongly affect the loading and/or proteolytic generation of an MHC-I-presented mature epitope peptide [44]. Collectively, our findings illustrate the flexibility of the HLA-A*02:01 molecule for the accommodation of a CMVp_495–503_ peptide with an N-extended endogenous sequence, even as fusion to an IgG Ab; this is not the case for N/C-extended peptides. Considering the size of a TEDbody, protrusion, rather than bulging, is a possible extension mechanism [45], although details of the mechanism remain to be determined via further structural studies.

For practical in vivo applications, a TEDbody was generated based on the full-length human IgG1/κ form but with an effector function-silenced Fc and a tumor tissue-homing ability by targeting of tumor-associated integrin αvβ3/αvβ5. Integrin αvβ3/αvβ5 are overexpressed on the surface of many types of epithelial tumor cells and tumor-associated blood vessels compared to normal cells and tissues [46]. However, any on-target/off-tumor adverse effects of TEDbody should be evaluated during clinical trials. Cytosolic processing-requiring TEDbody-mediated CMV-pMHCI presentation proved to be specific to tumor cells expressing both the tumor-associated membrane receptor (integrin αvβ5, for cytosolic access) and the HLA-A*02:01 allele (for epitope presentation), consequently confirming target cell specificity of the TEDbody. Moreover, systemic injection of inCT†CMVp_480–516_ into human tumor-bearing immunodeficient mice in our study marked a substantial proportion of the target tumor cells with CMV-pMHCI, thereby demonstrating the feasibility of converting tumor cells in vivo into CMV-infected cells, which renders the tumor susceptible to lysis by transferred CMVp-CTLs for attaining substantial antitumor effects. Furthermore, the in vivo TEDbody-mediated CMV-pMHCI presentation on target tumors seemed to serve as persistent antigenic stimulation of tumor-infiltrating CMVp-CTLs by augmenting their tumor infiltration and cytotoxicity in the TME.

Here, a combination therapy consisting of a TEDbody with an anti-OX40 agonistic Ab, but not with an anti-PD1 antagonistic Ab, yielded more potent TGI (than TEDbody monotherapy did) by enhancing the expansion and cytotoxic effector function of adoptively transferred CMVp-CTLs in the TME of immunodeficient mouse models. This evidence is in agreement with some reports on immune responses of murine CTLs against mouse CMV infection, wherein the proliferation and effector function of memory inflation-associated CTLs generated by low-level persistent CMV infection were promoted by OX40 costimulation [47, 48] but were retained independently of PD1 expression [49]. Our results suggest that antigenic stimulation by TEDbody-mediated CMV-pMHCI presentation maintains the effector memory phenotype of CMVp-CTLs, whereas the additional OX40 costimulation by the anti-OX40 agonistic Ab reinforces the expansion and cytotoxic effector function in the TME, thereby potentiating the antitumor action in comparison with TEDbody monotherapy. Our results also suggest that the PD1–PD-L1 immune checkpoint axis is not strongly associated with dysfunction of effector memory CMVp-CTLs [49]. On the other hand, simultaneous blockade of PD1 with other inhibitory receptors might be required to elicit strong antitumor responses of CMVp-CTLs. Overall, our data suggest that a combination with an anti-OX40 agonistic Ab, rather than PD1 blockade, is a potentially suitable strategy for improving clinical responses when CMVp-CTLs are repurposed against tumors.

Although CMV is not considered as an oncogenic virus, CMV antigens and nucleic acids have been detected prevalently in patients with a variety of cancer types, particularly glioblastoma [50]. This provides a rationale for adoptive transfer of ex vivo-expanded autologous CMV-specific CTLs to patients with CMV-infected tumors as cancer immunotherapy, which prolonged progression-free survival of some patients with recurrent glioblastoma [24, 50]. Mimicking a viral infection, specifically in tumor cells, with the aim of harnessing pre-existing virus-specific CTLs to attack the tumor, may offer an alternative to cancer immunotherapies involving neoantigen-based cancer vaccines [7]. Most neoantigens are tumor type-specific and/or patient-specific and require a personalized vaccine approach [1, 2], but antigens from viruses that commonly infect humans are likely to be shared among individuals, thus enabling the development of universal off-the-shelf cancer vaccines. Clinical relevance of the proposed TEDbody lies in the delivery of the immunodominant CTL epitope (CMVp_495–503_) for presentation on the most prevalent MHC-I molecule—HLA-A*02:01 (30–50% prevalence in the human population, depending on ethnicity)—according to the following facts: i) the widespread infection of adults by CMV (60–90% of the population), and ii) the unique properties of pre-existing CMVp-CTLs such as the high functional competence (mainly T_EM_ and T_EMRA_) and abundance (up to ~ 11% of CD8^+^ T cells) [9, 23]. Accordingly, the CMVp_495–503_-armed TEDbody is applicable to approximately 18–45% of cancer patients (in our study, 37.3% CMVp-CTL-positive PBMCs were detected in 28 out of 75 donors). In addition, given that viral infections are estimated to account for up to 20% of all cancer cases worldwide [51], the TEDbody technology may gain high popularity if it is extended to various cancer types. Nonetheless, there are major challenges for practical uses of TEDbodies against tumors, such as the following: presence of thousands of MHC-I allelic variants within the human population, and MHC-I loss or downregulation in tumor cells [52]. These challenges might be addressed by i) the development of a TEDbody carrying multiple epitope peptides to broaden the range of cancer patients that can be treated with this modality, and ii) combination therapy with either a cytokine (e.g., IFN-γ) or an appropriate chemical agent to restore MHC-I expression on tumor cells [52, 53].

## Conclusion

In conclusion, our study offers an effective technology for MHC-I antigen cytosolic delivery called TEDbody, which may help to develop a therapeutic cancer vaccine that delivers a viral CTL epitope directly into a target tumor, thereby making the tumor recognizable and killable by pre-existing antiviral CTLs in patients. Our approach requires information on immunogenic MHC-I-restricted viral epitopes and antiviral CTL immunophenotypes. The TEDbody technology can be utilized for cytosolic delivery of other (nonviral) MHC-I antigens, such as tumor neoantigens, into tumors lacking tumor antigens suitable for immunotherapy and has good potential for expanding the current arsenal of cancer immunotherapies.

## Supplementary Information

**Additional file 1: Figure S1.** SDS-PAGE analysis of the TEDbodies carrying various CMVp_495-503_-encompassing peptides. **Figure S2.** Cell surface expression levels of HLA-A*02, integrin αvβ3, integrin αvβ5, and PD-L1 in human cancer cell lines, as analyzed by flow cytometry. **Figure S3.** Flow cytometric determination of cell surface CMV-pMHCI presentation induced by a synthetic peptide (A) or TEDbody and control Ab (B). **Figure S4.** Prevalence of CMVp-CTLs among PBMCs before and after ex vivo expansion with the CMVp_495-503_ peptide. **Figure S5.** Immunophenotyping of CMVp-CTLs among PBMCs before and after ex vivo expansion with the CMVp_495-503_ peptide. **Figure S6.** Construction of TAP1 knockout MDA-MB-231 cells and MDA-MB-231-EGFP cells. **Figure S7.** Additional images of IHC detection of CMV-pMHCI (red) on MDA-MB-231 tumor tissues, the representative of which is shown in Fig. 3A (A) and Fig. 3E (B). **Figure S8.** The TEDbody suppresses in vivo growth of human tumor xenografts in immunodeficient NSG mice, as described in Fig. 3D. **Figure S9.** Binding specificity of the anti-OX40 agonistic 1166/1167 Ab and anti-PD1 antagonistic Ab (pembrolizumab), constructed and used in this study, respectively, to the surface-expressed antigen. **Figure S10.** In vivo antitumor efficacy of the inCT†CMVp_480-516_ TEDbody, combined with either the anti-OX40 1166/1167 Ab or anti-PD1 pembrolizumab, in NSG mice harboring preestablished MDA-MB-231 orthotopic tumor xenografts, as described in Fig. 4A. **Table S1.** List of synthesized peptides, used in this study. **Table S2.** List of resources (antibodies, recombinant proteins, and chemicals), used in this study. **Table S3.** Prevalence rates of CMVp-CTLs among PBMCs from HLA-A*02-positive healthy donors. **Table S4.** Characterization of CMVp-CTLs before and after ex vivo expansion with the CMVp_495-503_ peptide.

## Data Availability

The datasets supporting the conclusions of this article are included within the article and its additional file. All materials underlying this study are available from the corresponding author on the basis of a material transfer agreement.

## Abbreviations

CTL: Cytotoxic CD8^+^ T cells
MHC-I: Major histocompatibility complex class I
CMV: Cytomegalovirus
pMHCI: Peptide–MHC-I complex
ER: Endoplasmic reticulum
Ab: Antibody
PBS: Phosphate-buffered saline
TAP: Transporter associated with antigen processing
IHC: Immunohistochemistry
PD1: Programmed cell death protein 1
PD-L1: Programmed death-ligand 1
EGFP: Enhanced green fluorescent protein
LDH: Lactate dehydrogenase
IFN-γ: Interferon gamma
LAG-3: Lymphocyte-activation gene 3
TIGIT: T cell Ig and ITIM domain

## References

[1] Leko V, Rosenberg SA (2020). Identifying and targeting human tumor antigens for T cell-based immunotherapy of solid tumors. Cancer Cell.

[2] Saxena M, van der Burg SH, Melief CJM, Bhardwaj N (2021). Therapeutic cancer vaccines. Nat Rev Cancer.

[3] Lang F, Schrors B, Lower M, Tureci O, Sahin U. Identification of neoantigens for individualized therapeutic cancer vaccines. Nat Rev Drug Discov. 2022;21(4):261‒82. 10.1038/s41573-021-00387-y PMC7612664 35105974

[4] Ott PA, Hu Z, Keskin DB, Shukla SA, Sun J, Bozym DJ, Zhang W, Luoma A, Giobbie-Hurder A, Peter L (2017). An immunogenic personal neoantigen vaccine for patients with melanoma. Nature.

[5] Simoni Y, Becht E, Fehlings M, Loh CY, Koo SL, Teng KWW, Yeong JPS, Nahar R, Zhang T, Kared H (2018). Bystander CD8(+) T cells are abundant and phenotypically distinct in human tumour infiltrates. Nature.

[6] Rosato PC, Wijeyesinghe S, Stolley JM, Nelson CE, Davis RL, Manlove LS, Pennell CA, Blazar BR, Chen CC, Geller MA (2019). Virus-specific memory T cells populate tumors and can be repurposed for tumor immunotherapy. Nat Commun.

[7] Gujar S, Pol JG, Kim Y, Kroemer G (2020). Repurposing CD8(+) T cell immunity against SARS-CoV-2 for cancer immunotherapy: a positive aspect of the COVID-19 pandemic?. Oncoimmunology.

[8] Fischer C, Munks MW, Hill AB, Kroczek RA, Bissinger S, Brand V, Schmittnaegel M, Imhof-Jung S, Hoffmann E, Herting F (2020). Vaccine-induced CD8 T cells are redirected with peptide-MHC class I-IgG antibody fusion proteins to eliminate tumor cells in vivo. MAbs.

[9] Millar DG, Ramjiawan RR, Kawaguchi K, Gupta N, Chen J, Zhang S, Nojiri T, Ho WW, Aoki S, Jung K (2020). Antibody-mediated delivery of viral epitopes to tumors harnesses CMV-specific T cells for cancer therapy. Nat Biotechnol.

[10] Kang TH, Ma B, Wang C, Wu TC, Hung CF (2013). Targeted coating with antigenic peptide renders tumor cells susceptible to CD8(+) T cell-mediated killing. Mol Ther.

[11] Sefrin JP, Hillringhaus L, Mundigl O, Mann K, Ziegler-Landesberger D, Seul H, Tabares G, Knoblauch D, Leinenbach A, Friligou I (1962). Sensitization of tumors for attack by virus-specific CD8+ T-cells through antibody-mediated delivery of immunogenic T-cell epitopes. Front Immunol.

[12] Blum JS, Wearsch PA, Cresswell P (2013). Pathways of antigen processing. Annu Rev Immunol.

[13] Kim JS, Park JY, Shin SM, Park SW, Jun SY, Hong JS, Choi DK, Kim YS (2018). Engineering of a tumor cell-specific, cytosol-penetrating antibody with high endosomal escape efficacy. Biochem Biophys Res Commun.

[14] Shin SM, Kim JS, Park SW, Jun SY, Kweon HJ, Choi DK, Lee D, Cho YB, Kim YS (2020). Direct targeting of oncogenic RAS mutants with a tumor-specific cytosol-penetrating antibody inhibits RAS mutant-driven tumor growth. Sci Adv.

[15] Shin SM, Choi DK, Jung K, Bae J, Kim JS, Park SW, Song KH, Kim YS (2017). Antibody targeting intracellular oncogenic Ras mutants exerts anti-tumour effects after systemic administration. Nat Commun.

[16] Cannon MJ, Schmid DS, Hyde TB (2010). Review of cytomegalovirus seroprevalence and demographic characteristics associated with infection. Rev Med Virol.

[17] Klenerman P, Oxenius A (2016). T cell responses to cytomegalovirus. Nat Rev Immunol.

[18] Khan N, Shariff N, Cobbold M, Bruton R, Ainsworth JA, Sinclair AJ, Nayak L, Moss PA (2002). Cytomegalovirus seropositivity drives the CD8 T cell repertoire toward greater clonality in healthy elderly individuals. J Immunol.

[19] Wills MR, Carmichael AJ, Mynard K, Jin X, Weekes MP, Plachter B, Sissons JG (1996). The human cytotoxic T-lymphocyte (CTL) response to cytomegalovirus is dominated by structural protein pp65: frequency, specificity, and T-cell receptor usage of pp65-specific CTL. J Virol.

[20] Reiser JB, Legoux F, Machillot P, Debeaupuis E, Le Moullac-Vaydie B, Chouquet A, Saulquin X, Bonneville M, Housset D (2009). Crystallization and preliminary X-ray crystallographic characterization of a public CMV-specific TCR in complex with its cognate antigen. Acta Crystallogr Sect F Struct Biol Cryst Commun.

[21] Hyun SJ, Sohn HJ, Lee HJ, Lee SD, Kim S, Sohn DH, Hong CH, Choi H, Cho HI, Kim TG (2017). Comprehensive analysis of cytomegalovirus pp65 antigen-specific CD8(+) T cell responses according to human leukocyte antigen class I Allotypes and Intraindividual dominance. Front Immunol.

[22] Gonzalez-Galarza FF, McCabe A, Santos E, Jones J, Takeshita L, Ortega-Rivera ND, Cid-Pavon GMD, Ramsbottom K, Ghattaoraya G, Alfirevic A (2020). Allele frequency net database (AFND) 2020 update: gold-standard data classification, open access genotype data and new query tools. Nucleic Acids Res.

[23] Schmittnaegel M, Levitsky V, Hoffmann E, Georges G, Mundigl O, Klein C, Knoetgen H (2015). Committing cytomegalovirus-specific CD8 T cells to eliminate tumor cells by bifunctional major histocompatibility class I antibody fusion molecules. Cancer Immunol Res.

[24] Schuessler A, Smith C, Beagley L, Boyle GM, Rehan S, Matthews K, Jones L, Crough T, Dasari V, Klein K (2014). Autologous T-cell therapy for cytomegalovirus as a consolidative treatment for recurrent glioblastoma. Cancer Res.

[25] Nair SK, De Leon G, Boczkowski D, Schmittling R, Xie W, Staats J, Liu R, Johnson LA, Weinhold K, Archer GE (2014). Recognition and killing of autologous, primary glioblastoma tumor cells by human cytomegalovirus pp65-specific cytotoxic T cells. Clin Cancer Res.

[26] Kim YJ, Baek DS, Lee S, Park D, Kang HN, Cho BC, Kim YS (2019). Dual-targeting of EGFR and Neuropilin-1 attenuates resistance to EGFR-targeted antibody therapy in KRAS-mutant non-small cell lung cancer. Cancer Lett.

[27] Lee SY, Ko DH, Son MJ, Kim JA, Jung K, Kim YS (2021). Affinity maturation of a T-cell receptor-like antibody specific for a cytomegalovirus pp65-derived peptide presented by HLA-A*02:01. Int J Mol Sci.

[28] Kvarnhammar AM, Veitonmaki N, Hagerbrand K, Dahlman A, Smith KE, Fritzell S, von Schantz L, Thagesson M, Werchau D, Smedenfors K (2019). The CTLA-4 x OX40 bispecific antibody ATOR-1015 induces anti-tumor effects through tumor-directed immune activation. J Immunother Cancer.

[29] Kim JE, Lee DH, Jung K, Kim EJ, Choi Y, Park HS, Kim YS (2021). Engineering of humanized antibodies against human interleukin 5 receptor alpha subunit that cause potent antibody-dependent cell-mediated cytotoxicity. Front Immunol.

[30] Hu Q, Ye X, Qu X, Cui D, Zhang L, Xu Z, Wan H, Zhang L, Tao W (2018). Discovery of a novel IL-15 based protein with improved developability and efficacy for cancer immunotherapy. Sci Rep.

[31] Jung K, Ha JH, Kim JE, Kim JA, Kim YJ, Kim CH, Kim YS (2018). Heterodimeric fc-fused IL12 shows potent antitumor activity by generating memory CD8(+) T cells. Oncoimmunology.

[32] Jung K, Kim JA, Kim YJ, Lee HW, Kim CH, Haam S, Kim YS (2020). A Neuropilin-1 antagonist exerts antitumor immunity by inhibiting the suppressive function of Intratumoral regulatory T cells. Cancer Immunol Res.

[33] Choi DK, Bae J, Shin SM, Shin JY, Kim S, Kim YS (2014). A general strategy for generating intact, full-length IgG antibodies that penetrate into the cytosol of living cells. MAbs.

[34] Arifin WN, Zahiruddin WM (2017). Sample size calculation in animal studies using resource equation approach. Malays J Med Sci.

[35] Schlothauer T, Herter S, Koller CF, Grau-Richards S, Steinhart V, Spick C, Kubbies M, Klein C, Umana P, Mossner E (2016). Novel human IgG1 and IgG4 fc-engineered antibodies with completely abolished immune effector functions. Protein Eng Des Sel.

[36] Eisen HN, Hou XH, Shen C, Wang K, Tanguturi VK, Smith C, Kozyrytska K, Nambiar L, McKinley CA, Chen J (2012). Promiscuous binding of extracellular peptides to cell surface class I MHC protein. Proc Natl Acad Sci U S A.

[37] Keskin DB, Reinhold B, Lee SY, Zhang G, Lank S, O'Connor DH, Berkowitz RS, Brusic V, Kim SJ, Reinherz EL (2011). Direct identification of an HPV-16 tumor antigen from cervical cancer biopsy specimens. Front Immunol.

[38] Rock KL, York IA, Goldberg AL (2004). Post-proteasomal antigen processing for major histocompatibility complex class I presentation. Nat Immunol.

[39] Urban S, Textoris-Taube K, Reimann B, Janek K, Dannenberg T, Ebstein F, Seifert C, Zhao F, Kessler JH, Halenius A (2012). The efficiency of human cytomegalovirus pp65(495-503) CD8+ T cell epitope generation is determined by the balanced activities of cytosolic and endoplasmic reticulum-resident peptidases. J Immunol.

[40] Poiret T, Axelsson-Robertson R, Remberger M, Luo XH, Rao M, Nagchowdhury A, Von Landenberg A, Ernberg I, Ringden O, Maeurer M (2018). Cytomegalovirus-specific CD8+ T-cells with different T-cell receptor affinities segregate T-cell phenotypes and correlate with chronic graft-versus-host disease in patients post-hematopoietic stem cell transplantation. Front Immunol.

[41] Maben Z, Arya R, Rane D, An WF, Metkar S, Hickey M, Bender S, Ali A, Nguyen TT, Evnouchidou I (2020). Discovery of selective inhibitors of endoplasmic reticulum aminopeptidase 1. J Med Chem.

[42] Pham CD, Woo MY, Kim YS, Park S, Kwon MH (2012). An anti-nucleic acid antibody delivers antigen to the cross-presentation pathway in dendritic cells and potentiates therapeutic antitumor effects. J Immunol.

[43] Remesh SG, Andreatta M, Ying G, Kaever T, Nielsen M, McMurtrey C, Hildebrand W, Peters B, Zajonc DM (2017). Unconventional peptide presentation by major histocompatibility complex (MHC) class I allele HLA-A*02:01: BREAKING CONFINEMENT. J Biol Chem.

[44] Mo AX, van Lelyveld SF, Craiu A, Rock KL (2000). Sequences that flank subdominant and cryptic epitopes influence the proteolytic generation of MHC class I-presented peptides. J Immunol.

[45] Stryhn A, Pedersen LO, Holm A, Buus S (2000). Longer peptide can be accommodated in the MHC class I binding site by a protrusion mechanism. Eur J Immunol.

[46] Nieberler M, Reuning U, Reichart F, Notni J, Wester HJ, Schwaiger M, et al. Exploring the role of RGD-recognizing Integrins in Cancer. Cancers (Basel). 2017;9(9):116. 10.3390/cancers9090116 PMC5615331 28869579

[47] Humphreys IR, Loewendorf A, de Trez C, Schneider K, Benedict CA, Munks MW, Ware CF, Croft M (2007). OX40 costimulation promotes persistence of cytomegalovirus-specific CD8 T cells: a CD4-dependent mechanism. J Immunol.

[48] Panagioti E, Boon L, Arens R, van der Burg SH (2017). Enforced OX40 stimulation empowers booster vaccines to induce effective CD4(+) and CD8(+) T cell responses against mouse cytomegalovirus infection. Front Immunol.

[49] Erkes DA, Smith CJ, Wilski NA, Caldeira-Dantas S, Mohgbeli T, Snyder CM (2017). Virus-specific CD8(+) T cells infiltrate melanoma lesions and retain function independently of PD-1 expression. J Immunol.

[50] Luo XH, Meng Q, Rao M, Liu Z, Paraschoudi G, Dodoo E, Maeurer M (2018). The impact of inflationary cytomegalovirus-specific memory T cells on anti-tumour immune responses in patients with cancer. Immunology.

[51] Morales-Sanchez A, Fuentes-Panana EM (2014). Human viruses and cancer. Viruses..

[52] Dhatchinamoorthy K, Colbert JD, Rock KL (2021). Cancer immune evasion through loss of MHC class I antigen presentation. Front Immunol.

[53] Wan S, Pestka S, Jubin RG, Lyu YL, Tsai YC, Liu LF (2012). Chemotherapeutics and radiation stimulate MHC class I expression through elevated interferon-beta signaling in breast cancer cells. PLoS One.

