# Supplementary material for: Antibody-mediated delivery of a viral MHC-I epitope into the cytosol of target tumor cells repurposes virus-specific CD8+ T cells for cancer immunotherapy
Source: Mol Cancer. 2022 Apr 22;21:102. doi: 10.1186/s12943-022-01574-0 (PMC9027861; doi:10.1186/s12943-022-01574-0)
Supplement: Supplementary file 1 — Additional file 1: Figure S1. SDS-PAGE analysis of the TEDbodies carrying various CMVp495-503-encompassing peptides. Figure S2. Cell surface expression levels of HLA-A*02, integrin αvβ3, integrin αvβ5, and PD-L1 in human cancer cell lines, as analyzed by flow cytometry. Figure S3. Flow cytometric determination of cell surface CMV-pMHCI presentation induced by a synthetic peptide (A) or TEDbody and control Ab (B). Figure S4. Prevalence of CMVp-CTLs among PBMCs before and after ex vivo expansion with the CMVp495-503 peptide. Figure S5. Immunophenotyping of CMVp-CTLs among PBMCs before and after ex vivo expansion with the CMVp495-503 peptide. Figure S6. Construction of TAP1 knockout MDA-MB-231 cells and MDA-MB-231-EGFP cells. Figure S7. Additional images of IHC detection of CMV-pMHCI (red) on MDA-MB-231 tumor tissues, the representative of which is shown in Fig. 3A (A) and Fig. 3E (B). Figure S8. The TEDbody suppresses in vivo growth of human tumor xenografts in immunodeficient NSG mice, as described in Fig. 3D. Figure S9. Binding specificity of the anti-OX40 agonistic 1166/1167 Ab and anti-PD1 antagonistic Ab (pembrolizumab), constructed and used in this study, respectively, to the surface-expressed antigen. Figure S10. In vivo antitumor efficacy of the inCT†CMVp480-516 TEDbody, combined with either the anti-OX40 1166/1167 Ab or anti-PD1 pembrolizumab, in NSG mice harboring preestablished MDA-MB-231 orthotopic tumor xenografts, as described in Fig. 4A. Table S1. List of synthesized peptides, used in this study. Table S2. List of resources (antibodies, recombinant proteins, and chemicals), used in this study. Table S3. Prevalence rates of CMVp-CTLs among PBMCs from HLA-A*02-positive healthy donors. Table S4. Characterization of CMVp-CTLs before and after ex vivo expansion with the CMVp495-503 peptide. [file 12943_2022_1574_MOESM1_ESM.pdf]

Supplementary information for

Antibody-mediated delivery of a viral MHC-I epitope into the cytosol of target tumor cells repurposes virus-specific CD8$^+$ T cells for cancer immunotherapy

Keunok Jung, Min-Jeong Son, Se-Young Lee, Jeong-Ah Kim, Deok-Han Ko, Sojung Yoo, Chul-Ho Kim, Yong-Sung Kim

This additional file includes:

Figs. S1 to S10
Tables S1 to S4

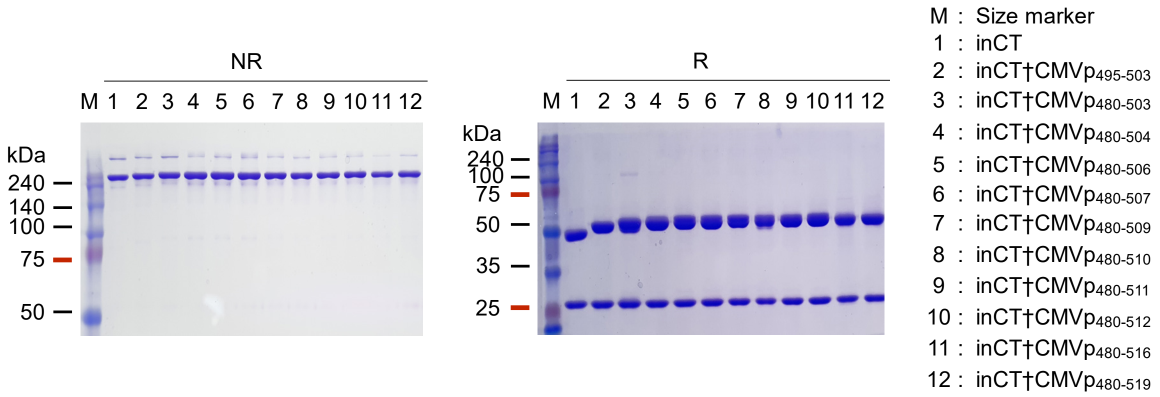

Figure S1. SDS-PAGE analysis of the TEDbodies carrying various $CMVp_{495\text{-}503}$-encompassing peptides.

TEDbodies, designed as shown in Fig. 1B, were expressed using a standard HEK293F transient-expression system. The purified TEDbody (each 5 µg) was analyzed by SDS-PAGE in a 10% gel under nonreducing conditions in the absence of the reducing agent dithiothreitol (DTT) (”NR”) (left panel), as well as by SDS-PAGE in a 12% gel under reducing conditions in the presence of 20 mM DTT (“R”) (right panel), and then was stained with Coomassie Brilliant Blue. The SDS-PAGE analysis revealed the presence of the heavy chain of a TEDbody with higher molecular weight than that of inCT due to the C terminus-fused peptide on the heavy chain. The inCT Ab was used as a control.

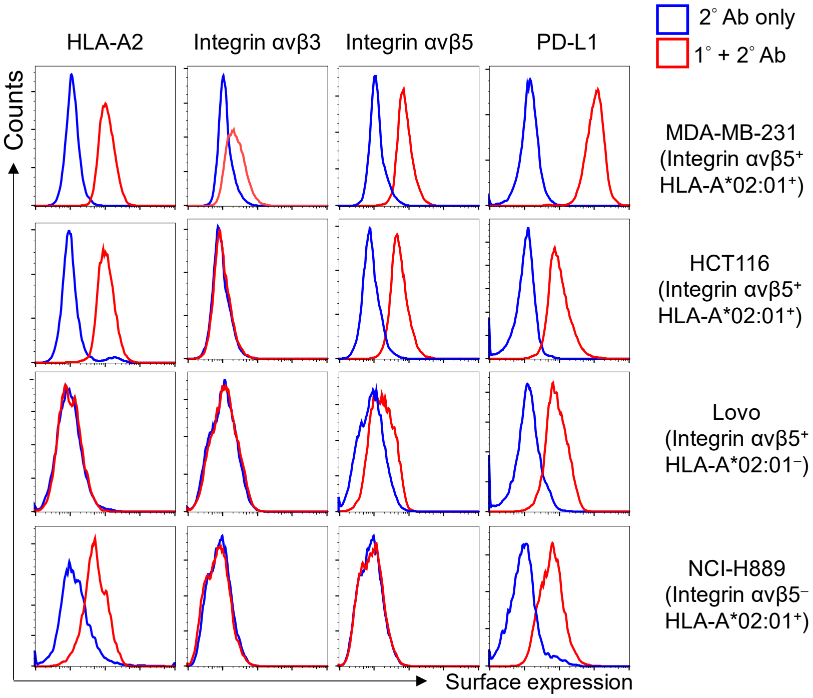

Figure S2. Cell surface expression levels of HLA-A*02, integrin αvβ3, integrin αvβ5, and PD-L1 in human cancer cell lines, as analyzed by flow cytometry.

Representative flow cytometric histograms of two independent experiments are shown. Based on these analyses, the cells were designated as follows: MDA-MB-231 cells as HLA-A*02:01^{+} integrin αvβ3/αvβ5^{+} PD-L1^{+}, HCT116 cells as HLA-A*02:01^{+} integrin αvβ5^{+} integrin αvβ3^{-} PD-L1^{+}, LoVo cells as HLA-A*02:01^{-} integrin αvβ5^{+} integrin αvβ3^{-} PD-L1^{+}, and NCI-H889 cells as HLA-A*02:01^{+} integrin αvβ3/αvβ5^{-} PD-L1^{+}.

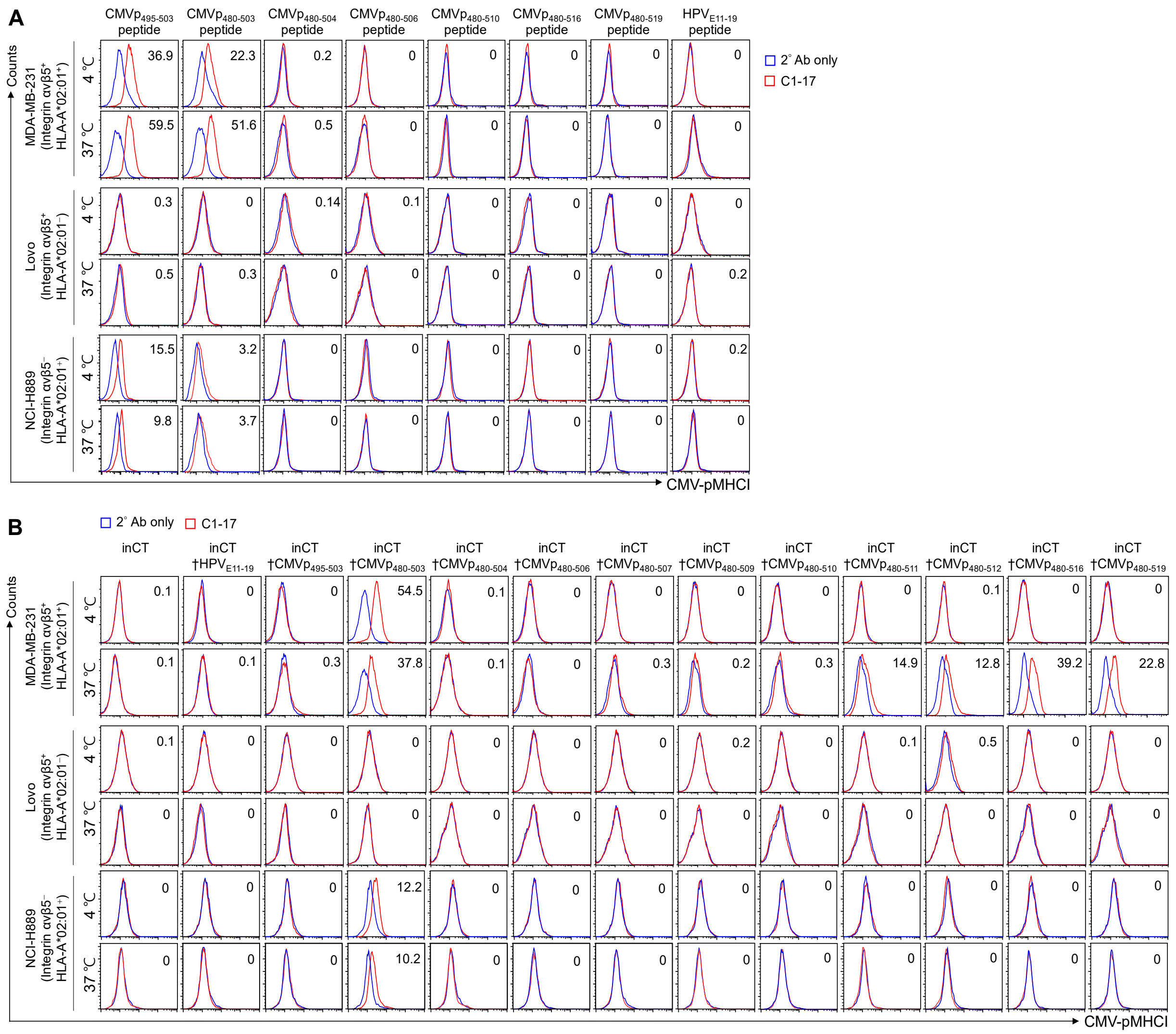

Figure S3. Flow cytometric determination of cell surface CMV-pMHCI presentation induced by a synthetic peptide (A) or TEDbody and control Ab (B).

A flow cytometric histogram of CMV-pMHCI display on the surface of HLA-A*02:01^{+} MDA-MB-231 cells and HLA-A*02:01^{-} LoVo cells, detected by the CMV-pMHCI-specific C1-17 Ab (red) in comparison with the control involving only the secondary Ab (blue). The cells were treated with the indicated synthetic peptide, TEDbody, or control Ab (4 µM) at 4°C for 3 h or at 37°C for 18 h, followed by flow cytometric analysis of the cell surface presentation of CMV-pMHCI. A representative histogram from at least three independent experiments is shown.

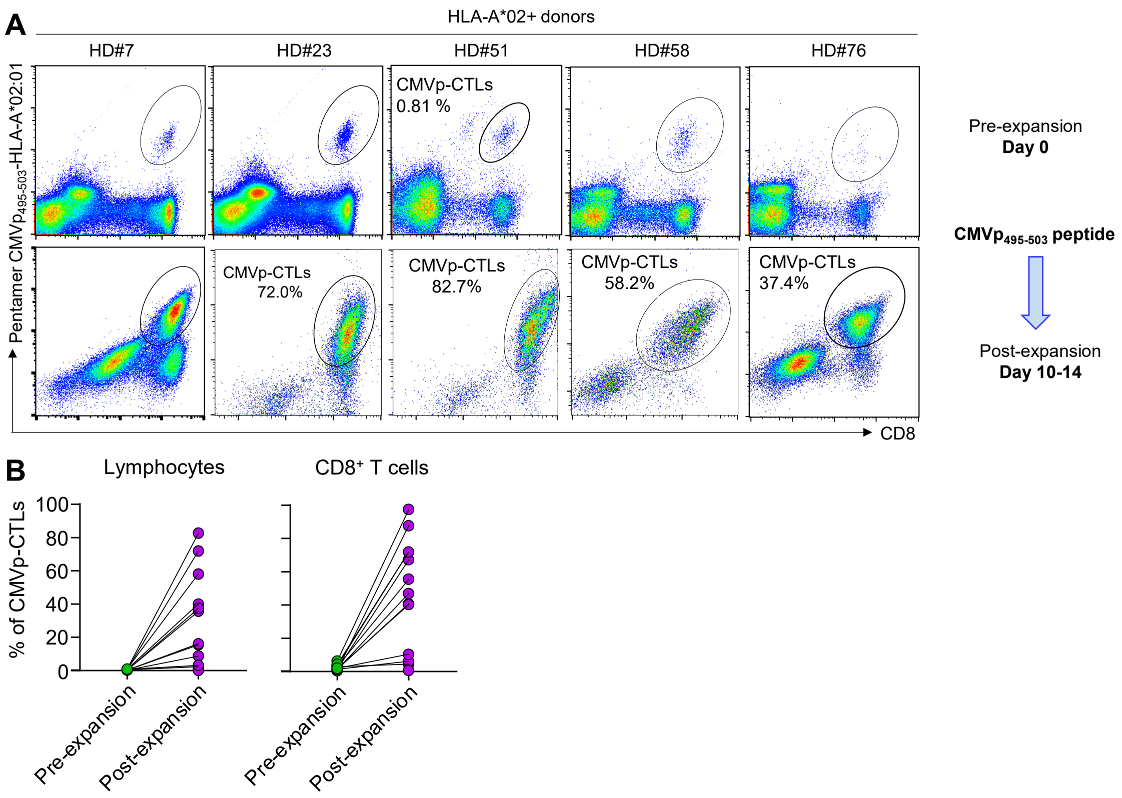

Figure S4. Prevalence of CMVp-CTLs among PBMCs before and after *ex vivo* expansion with the CMV$p_{495\text{-}503}$ peptide.

(**A** and **B**) Representative flow cytometry dot plots showing prevalence rates of CMVp-CTLs (n = 5) (A) and the pairwise analysis (n = 12) (B) of PBMCs from HLA-A*02-positive, CMVp-CTL-seropositive healthy donors, before and after the *ex vivo* expansion with the synthetic CMV$p_{495\text{-}503}$ peptide (5 µM) and cytokines for 14 to 18 days, as analyzed via flow cytometry by double staining with an anti-CD8 Ab and CMV$p_{495\text{-}503}$-HLA-A*02:01 pentamer and the gating settings shown in the plot. In (A), PBMCs were defined as CMVp-CTL-positive when the prevalence of CMVp-CTLs (i.e., double-positive cells stained with the anti-CD8 Ab and PE-conjugated CMV$p_{495\text{-}503}$-HLA-A*02:01 pentamer) is more than 0.1% of $CD8^+$ T cells among PBMCs ($\geq 2.0 \times 10^5$ events were collected using the FACSCalibur flow cytometer). In (B), each symbol represents a value obtained from an individual donor. In total, PBMCs from 75 donors were analyzed. Forty-five out of 75 donors were positive for HLA-A*02, and 28 out of 45 HLA-A*02-positive donors were positive for CMVp-CTLs. Please see Table S2 for details.

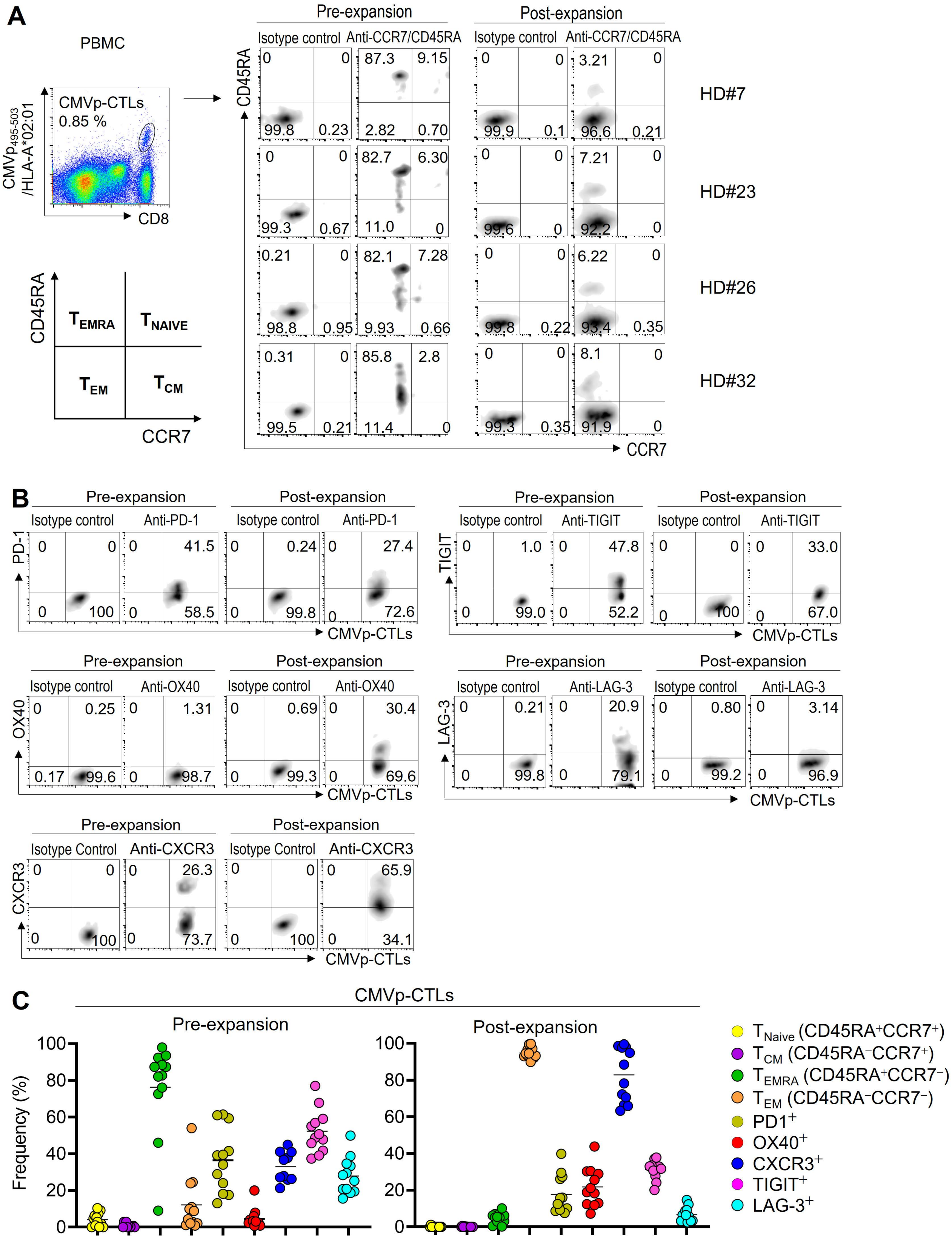

Figure S5. Immunophenotyping of CMVp-CTLs among PBMCs before and after *ex vivo* expansion with the CMVp$_{495\text{-}503}$ peptide.

(**A**) A representative flow cytometry dot plot showing the memory phenotype analysis of CMVp-CTLs among PBMCs from four donors, based on CCR7 and CD45RA expression on the cells. The inset is a schematic diagram categorizing CMVp-CTLs into the following four subsets: $CCR7^{+}CD45RA^{+}$ naïve (T_{Naive}) cells, $CCR7^{+}CD45RA^{-}$ central memory (T_{CM}) cells, $CCR7^{-}CD45RA^{-}$ effector memory (T_{EM}) cells, and $CCR7^{-}CD45RA^{+}$ terminally differentiated effector memory (T_{EMRA}) cells.
(B) A representative flow cytometry dot plot showing the expression of PD1, OX40, CXCR3, TIGIT, and LAG-3 among the CMVp-CTLs from PBMCs before and after the *ex vivo* expansion with the synthetic $CMVp_{495-503}$ peptide, as described in Fig. S4A.
(C) Prevalence rates of the four $CD8^{+}$ T cell subsets and cells expressing PD1, OX40, CXCR3, TIGIT, and LAG-3 among the CMVp-CTLs from PBMCs. The detection limit for the expression of PD1, OX40, CXCR3, TIGIT, and LAG-3 was set to 0.01% in the flow cytometric analysis. Midlines represent the mean values. 12 CMVp-CTL-positive PBMCs, before and after the *ex vivo* expansion with the synthetic $CMVp_{495-503}$ peptide, as described in Fig. S4A, were analyzed by flow cytometry. Each symbol represents a value obtained from an individual donor. See Table S4 for the details.

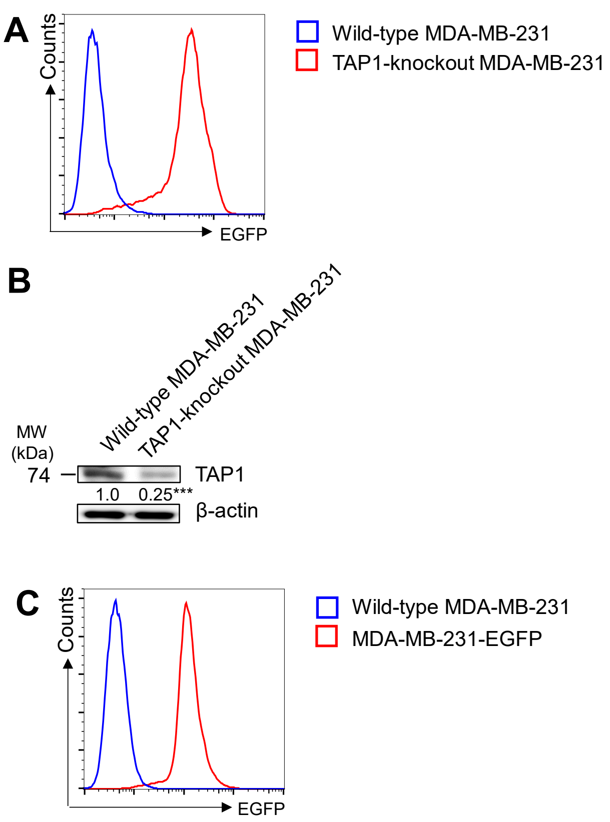

Figure S6. Construction of TAP1 knockout MDA-MB-231 cells and MDA-MB-231-EGFP cells.

(**A**) A flow cytometry histogram intended to assess sgRNA and Cas9 expression in an obtained stable TAP1 knockout MDA-MB-231 cell line (red line), in comparison with wild-type MDA-MB-231 cells (blue line), based on EGFP expression analysis. TAP1 knockout MDA-MB-231 cells were generated using modified all-in-one lentiviral vector eSpCas9-LentiCRISPIR v2-TAP1, containing a TAP1-targeting sgRNA sequence, eSpCas9 for enhanced target specificity, and *EGFP* as a selection marker.

(**B**) Representative western blots showing TAP1 deletion efficiency in TAP1-knockout MDA-MB-231 cells compared with wild-type MDA-MB-231 cells. TAP1 was detected by anti-TAP1 Ab (clone B-8). The number below the panel indicates the mean value (n = 2) of band intensity relative to that of wild-type MDA-MB-231 cells after normalization of the band intensity to that of respective β-actin. ***P < 0.001 vs. wild-type MDA-MB-231 cells. A standard procedure for western blotting was performed. Equal amount of lysates was analyzed by western blotting, with β-actin serving as a loading control. Band intensities were quantified using the ImageJ software.

(**C**) A flow cytometric histogram for assessing EGFP expression in the newly established stable MDA-MB-231-EGFP cells (red line), compared to wild-type MDA-MB-231 cells (blue line).

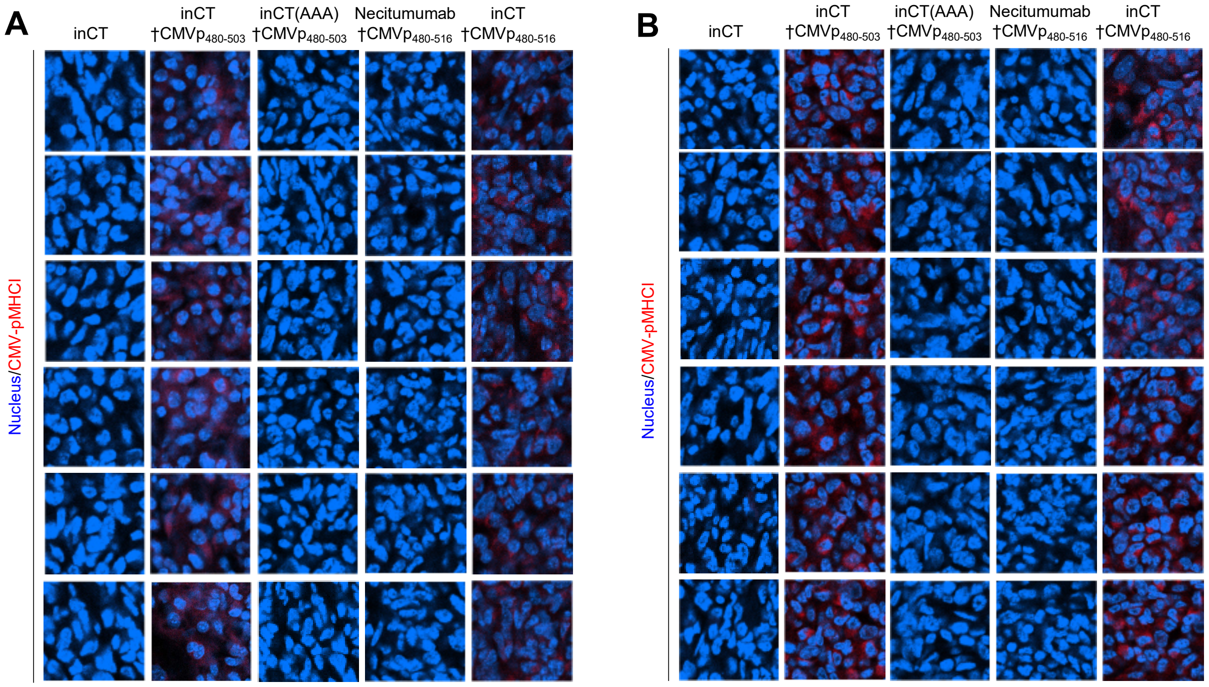

Figure S7. Additional images of IHC detection of CMV-pMHCI (red) on MDA-MB-231 tumor tissues, the representative of which is shown in Fig. 3A (A) and Fig. 3E (B).

(A) Images for the tumor tissues excised from NSG mice bearing a preestablished MDA-MB-231 cell-derived tumor xenograft (100–120 mm^3), 24 h after a single i.p. injection of the indicated TEDbody or control Ab (20 mpk), as described in Fig. 3A.

(B) Images for the tumor tissues excised from mice on day 3 after the last treatment, as described in Fig. 3C. The quantification of the CMV-pMHCI-positive areas (red fluorescence intensity) is shown in Fig. 3E.

In (A) and (B), nuclei were stained with Hoechst 33342 (blue). Scale bar: 20 μm.

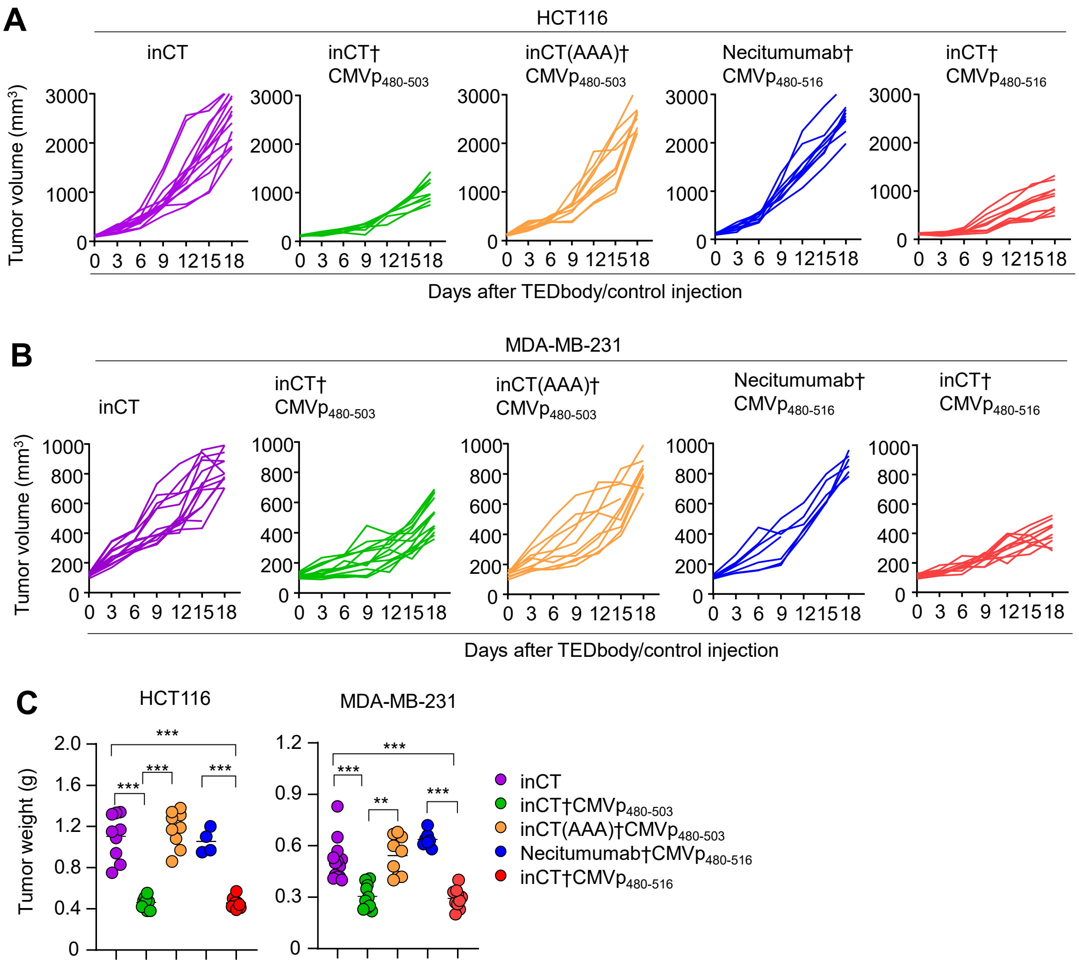

Figure S8. The TEDbody suppresses *in vivo* growth of human tumor xenografts in immunodeficient NSG mice, as described in Fig. 3D.

(**A** and **B**) Tumor growth profiles of individual mice bearing an HCT116 (A) or MDA-MB-231 tumor xenograft (B), treated as described in Fig. 3D. Each curve represents a tumor growth curve, constructed using tumor volume data from each mouse. The data from two independent experiments were pooled.

(**C**) In each treatment group, representative weight of individual tumors excised from mice on day 3 after the last treatment, as described in Fig. 3D. Each symbol represents a value obtained from an individual mouse. Midlines represent the mean values. $^{**}P < 0.01$ and $^{***}P < 0.001$, a significant difference from the inCT group or between the indicated groups.

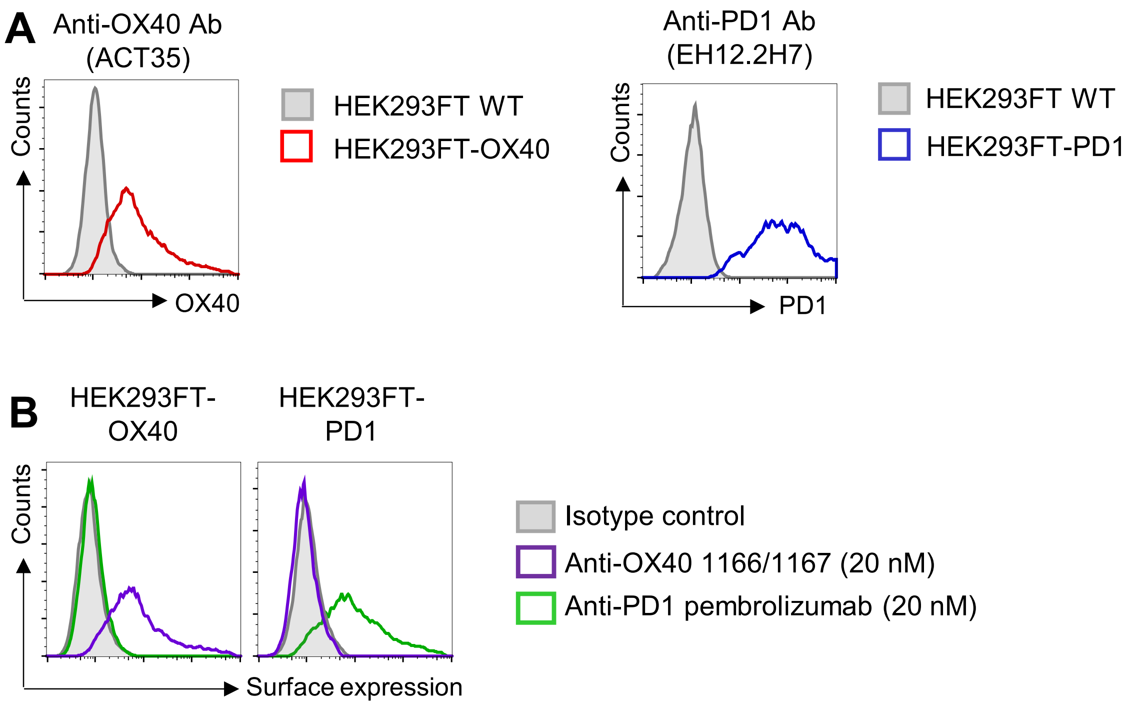

Figure S9. Binding specificity of the anti-OX40 agonistic 1166/1167 Ab and anti-PD1 antagonistic Ab (pembrolizumab), constructed and used in this study, respectively, to the surface-expressed antigen.

(**A**) Flow cytometric analysis of the surface expression of OX40 with a commercial anti-OX40 Ab (ACT365, Invitrogen) on HEK293FT wild type (H293T WT) cells and HEK293FT cells transiently expressing OX40 (HEK293FT-OX40), or the surface expression of PD1 using a commercial anti-PD1 Ab (EH12.2H7, Biolegend) on HEK293FT WT cells and HEK293FT cells transiently expressing PD1 (HEK293FT-PD1).

(**B**) Flow cytometric analysis based on anti-OX40 1166/1167 Ab (left) and anti-PD1 Ab (pembrolizumab, right) binding to HEK293FT-OX40 and HEK293FT-PD1 cells, respectively.

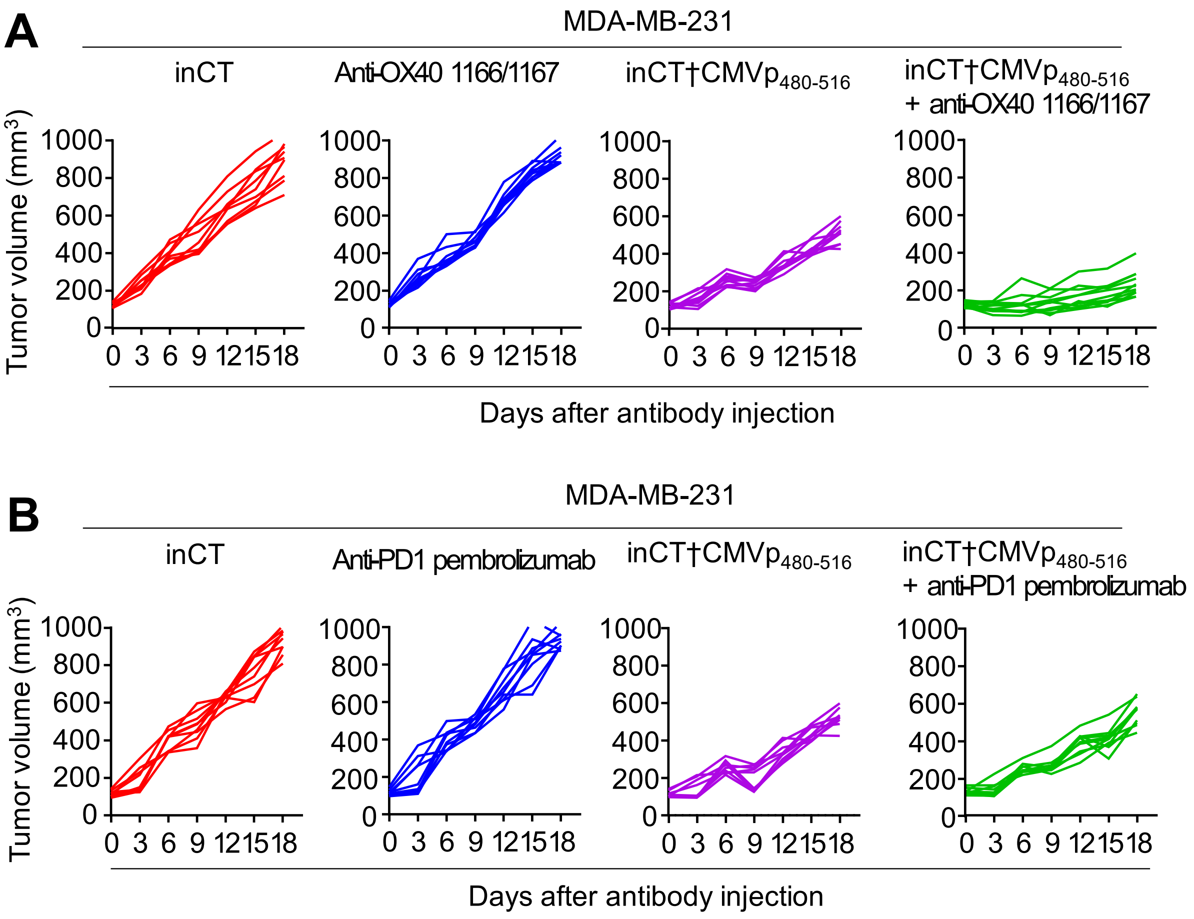

Figure S10. *In vivo* antitumor efficacy of the inCT†CMVp480-516 TEDbody, combined with either the anti-OX40 1166/1167 Ab or anti-PD1 pembrolizumab, in NSG mice harboring preestablished MDA-MB-231 orthotopic tumor xenografts, as described in Fig. 4A.
(**A** and **B**) Tumor growth profiles of individual mice bearing an MDA-MB-231 tumor, treated with the combination described in Fig. 4A. Each curve represents a tumor growth curve, constructed based on tumor volume data from an individual mouse. The data from two independent experiments were pooled.

Table S1. List of synthesized peptides, used in this study.

Peptide	Sequence
$CMVp_{495\text{-}503}$	^{495}NLVPMVATV^{503}
$CMVp_{480\text{-}503}$	^{480}AVFTWPPWQAGILARNLVPMVATV^{503}
$CMVp_{480\text{-}504}$	^{480}AVFTWPPWQAGILARNLVPMVATVQ^{504}
$CMVp_{480\text{-}506}$	^{480}AVFTWPPWQAGILARNLVPMVATVQGQ^{506}
$CMVp_{480\text{-}510}$	^{480}AVFTWPPWQAGILARNLVPMVATVQGQNLKY^{510}
$CMVp_{480\text{-}516}$	^{480}AVFTWPPWQAGILARNLVPMVATVQGQNLKYQEFFWD^{516}
$CMVp_{480\text{-}519}$	^{480}AVFTWPPWQAGILAR NLVPMVATVQGQNLKYQEFFWDAND^{519}
$HPV_{E11\text{-}19}$	^{11}YMLDLQPET^{19}

Table S2. List of resources (antibodies, recombinant proteins, and chemicals), used in this study.

Antibodies or Reagents	Company	Catalog no.
**Antibodies**		
Anti-Human IFN-gamma monoclonal Antibody (4S.B3)-FITC	Invitrogen	Cat. # 11-7319-82, RRID : AB_465415
Anti-Human CD8a monoclonal Antibody (SK1)-PerCP eFlour710	Invitrogen	Cat. # 46-0087-42, RRID : AB_1834411
Anti-Human CD8a monoclonal antibody (RPA-T8)-APC	Invitrogen	Cat. # 17-0088-42, RRID : AB_10669564
Anti-Human CD62L (L-Selectin) monoclonal Antibody (DREG56)-APC	Invitrogen	Cat. # 17-0629-41, RRID : AB_10671132
Anti-Human CD45RA monoclonal antibody (HI100) -FITC	Invitrogen	Cat. #11-0458-42, RRID : AB_940360
Anti-Human CD107a (LAMP-1) monoclonal Antibody(eBioH4A3)-FITC	Invitrogen	Cat. # 11-1079-42, AB_10853196
Anti-Human CCR7monoclonal antibody (3D12) -PerCP-eFluor 710	Invitrogen	Cat# 46-1979-42, RRID : AB_10853814
Anti-Human CD134(OX40) monoclonal antibody (ACT35)-FITC	Invitrogen	Cat. # 11-1347-42, RRID : AB_10597448
Anti-Human CD69 monoclonal antibody (FN50)-FITC	Invitrogen	Cat. # 11-0699-42, RRID : AB_10853975
Goat polyclonal anti-mouse IgG antibody, Alexa Fluor 488-conjugated	Invitrogen	Cat. # A-11001, RRID: AB_2534069
Mouse monoclonal anti-human integrin αvβ3 antibody (23C6)	R&D Systems	Cat. # MAB3050, RRID: AB_2128187
Mouse monoclonal anti-human integrin αvβ5 antibody (P5H9)	R&D Systems	Cat. # MAB2528, RRID: AB_2280706
Anti-Human CD279 (PD1) monoclonal Antibody (EH12.2H7)-APC	Biolegend	Cat. # 329908, RRID : AB_940475
Anti-Human CD274 (PD-L1) monoclonal Antibody (B7-H1)-APC	Biolegend	Cat. # 329708, RRID : AB_940360
Anti-Human CD223 (LAG-3) monoclonal Antibody (7H2C65)-FITC	Biolegend	Cat. # 369210, RRID : AB_2716129
Anti-Human TIGIT (VSTM3) monoclonal Antibody (A15153G)-APC	Biolegend	Cat. # 372706, RRID : AB_2632732
Anti-Human HLA-A2 monoclonal Antibody(BB7.2)-PE	Santa cruz Biotechnology	Cat# sc-32236-PE
Anti-human EEA1 monoclonal antibody (281.7)	Santa cruz Biotechnology	Cat. # sc-53939
Anti-human FTCD monoclonal antibody (58K-9)	Santa cruz Biotechnology	Cat. # sc-53128
Anti-TAP1 antibody (B-8)	Santa cruz Biotechnology	Cat. # sc-376796
CMV pp65$_{495-503}$/HLA-A*02:01 pentamer-PE	Proimmune	Cat. # F008-2A
Human IFN-gamma ELISA Kit	Thermo Fisher Scientific	Cat. # 88-7316-88
**Chemicals and Recombinant Proteins**		
Streptavidin-conjugated R-phycoerythrin (SA-PE)	Thermo Fisher Scientific	Cat. # S21388
MG132	Thermo Fisher Scientific	Cat. # M7449
Dylight 550 Antibody labeling Kit	Thermo Fisher Scientific	Cat. # 84530
ERAP1-IN-1	Chem Scene	Cat. # CS-0112138
Recombinant Human IL-2	Peprotech	Cat. # 200-02
Lipofectamine 3000	Invitrogen	Cat. # L3000008
Isoflurane	Piramal Critical Care	Cat. # 66794-017-25
Brefeldin A	Thermo Fisher Scientific	Cat. # 00-4506-51
PKH26	Sigma	Cat. # MINI26-1KT
Poly-L-lysine solution	Sigma	Cat. # P8920
Matrigel Basement Membrane Matrix	Corning	Cat. # 354234
Fluorescence Mounting Medium	Dako	Cat. # 3023

Table S3. Prevalence rates of CMVp-CTLs among PBMCs from HLA-A*02-positive healthy donors.

Donor	HLA-A*02 expression	CMVp-CTL (%)		Donor	HLA-A*02 expression	CMVp-CTL (%)	
		out of PBMCs	out of $CD8^+$ T cells			out of PBMCs	out of $CD8^+$ T cells
HD#1	-			HD#39	–		
HD#2	-			HD#40	–		
HD#3	+	–	–	HD#41	–		
HD#4	-			HD#42	+	0.18	0.58
HD#5	-			HD#43	–		
HD#6	+	0.07	0.36	HD#44	+	–	–
HD#7	+	0.52	1.7	HD#45	+	0.71	2.4
HD#8	+	–	–	HD#46	–		
HD#9	-			HD#47	–		
HD#10	-			HD#48	–		
HD#11	-			HD#49	–		
HD#12	+	0.04	0.14	HD#50	+	-	-
HD#13	+	0.57	3.03	HD#51	+	0.73	1.0
HD#14	+	0.13	0.6	HD#52	–		
HD#15	-			HD#53	+	0.079	0.49
HD#16	-			HD#54	–		
HD#17	+	0.11	0.66	HD#55	–		
HD#18	+	0.14	0.64	HD#56	+	0.07	0.19
HD#19	+	0.12	0.74	HD#57	–		
HD#20	-			HD#58	+	1.01	3.64
HD#21	+	0.72	2.46	HD#59	–		
HD#22	+	–	–	HD#60	+	–	–
HD#23	+	0.72	5.34	HD#61	+	0.04	0.26
HD#24	-			HD#62	+	–	–
HD#25	+	–	–	HD#63	+	–	–
HD#26	+	0.85	5.06	HD#64	+	–	–
HD#27	+	0.39	1.25	HD#65	–		

HD#28	+	0.08	0.37	HD#66	+	0.36	1.61
HD#29	+	–	–	HD#67	+	0.07	0.40
HD#30	–			HD#68	–		
HD#31	+	–	–	HD#69	+	–	–
HD#32	+	0.3	0.94	HD#70	+	–	–
HD#33	+	0.13	0.57	HD#71	+	0.15	1.17
HD#34	+	–	–	HD#72	+	0.8	3.6
HD#35	+	–	–	HD#73	+	0.29	1.85
HD#36	+	–	–	HD#74	–	–	–
HD#37	–	–	–	HD#75	+	0.21	1.45
HD#38	–						

In total, PBMCs from 75 healthy donors were analyzed. Forty-five out of 75 donors were positive for HLA-A*02. The 45 samples of PBMCs from the HLA-A*02-positive donors were analyzed via flow cytometry by double staining with the anti-CD8 Ab and PE-conjugated $CMVp_{495-503}$-HLA-A*02:01 pentamer, to determine the percentages of CMVp-CTLs among PBMCs and $CD8^+$ T cells, as shown in Fig. S4A. PBMCs were defined as CMVp-CTL-positive when the prevalence of CMVp-CTLs (i.e., double-positive cells stained with the anti-CD8 Ab and PE-conjugated $CMVp_{495-503}$-HLA-A*02:01 pentamer) was more than 0.1% of $CD8^+$ T cells among PBMCs ($\geq 2.0\times10^5$ cells counted). Twenty-eight samples out of 45 samples of PBMCs from 45 HLA-A*02-positive donors were CMVp-CTL-positive.

Table S4. Characterization of CMVp-CTLs before and after *ex vivo* expansion with the $CMVp_{495\text{-}503}$ peptide

			Donors											
			HD#7	HD#21	HD#23	HD#26	HD#32	HD#51	HD#58	HD#71	HD#72	HD#73	HD#75	HD#76
**Pre-expansion**	**CMVp-CTLs (%)**	Total	0.31	0.72	0.52	0.61	0.42	0.81	0.79	0.15	0.8	0.29	0.21	0.15
		$CD8^+$ T	1.42	2.46	2.94	2.79	1.44	1	5.92	1.17	3.6	1.85	1.45	0.79
	**Memory phenotype (% of CMVp-CTLs)**	T_{Naive}	9.15	4.9	6.3	7.28	0	5.33	10.4	1.89	2.9	0.98	0	0
		T_{CM}	0.7	0.7	0	0.66	0	0	0.29	0.94	2.9	2.94	0	0
		T_{EM}	2.82	2.1	11.0	9.93	54.0	1.18	0.86	24.5	4.35	8.82	23.9	2.05
		T_{EMRA}	87.3	92.3	82.7	82.1	46.0	93.5	88.4	72.6	8.99	87.3	76.1	97.9
	**Immune checkpoint expression (% of CMVp-CTLs)**	PD1	41.5	17.6	39.2	34.1	39.5	24	59.3	13.1	18.2	61.4	61	35
		TIGIT	77.0	55.0	56.9	41.7	59.1	97.8	47.8	48.7	50.7	37.4	39.1	45.8
		LAG-3	33.3	38.7	20.8	19.5	15.6	18.7	20.9	25.3	29.5	28.4	49.9	34.7
		OX40	4.08	2.64	2.35	7.94	2.47	6	0.88	3.27	0.7	3.12	20	12.5
	**Pro-inflammatory chemokine receptor (% of CMVp-CTLs)**	CXCR3	41.1	37.8	28.6	44.9	25.9	40.9	36.7	21.3	27.9	26.3	27.1	45.2
**Post-expansion**	**CMVp-CTLs (%)**	Total	35.6	11.9	72	1.06	2.35	82.7	58.2	16.5	40	16.2	8.78	37.4
		$CD8^+$ T	67.3	20.7	95.3	4.47	6.06	97.5	93.5	47	72	40.3	40.7	87.8
	**Memory phenotype (% of CMVp-CTLs)**	T_{Naive}	0	0	0	0	0	0	0.37	0	1.13	0	0	0
		T_{CM}	0.21	0	0	0.35	0	0	0	0	0.53	0	0	0
		T_{EM}	96.6	93.0	92.2	93.4	93.8	89.9	94.51	99.5	93.3	97.1	94.5	99.8
		T_{EMRA}	3.21	7.0	7.21	6.22	6.2	10.1	5.13	0.5	5.01	2.9	5.5	0.22
	**Immune checkpoint expression (% of CMVp-CTLs)**	PD1	10.1	10.1	26.1	27.4	8.67	16	15.8	7.58	9.34	29.5	39.8	12.5
		TIGIT	37.1	32.5	33.4	27.7	32.9	33.5	33.0	30.7	37.8	24.2	20.1	31.7
		LAG-3	6.56	3.82	5.59	3.4	3.25	2.53	3.14	14.7	8.8	12.3	9.22	6.5
		OX40	18.9	12.4	30.3	30.4	13	11.9	7.26	18.3	21.2	28.9	25.6	43.8
	**Pro-inflammatory chemokine receptor (% of CMVp-CTLs)**	CXCR3	98.6	98	97.7	94.9	66.4	88.4	63.3	72.3	78.2	65.9	99.5	70.7

CMVp-CTLs were analyzed by flow cytometry for the memory phenotypes, based on the surface expression of CCR7 and CD45RA in the four subsets (T_{Naive}, T_{CM}, T_{EM}, and T_{EMRA}), as presented in Fig. S5A, and for the surface expression of PD1, OX40, CXCR3, TIGIT, and LAG-3, detected with appropriate primary and secondary Abs, as presented in Fig. S5B.
